# CD1d Expression in Paneth Cells and Rat Exocrine Pancreas Revealed by Novel Monoclonal Antibodies Which Differentially Affect NKT Cell Activation

**DOI:** 10.1371/journal.pone.0013089

**Published:** 2010-09-30

**Authors:** Elisa Monzon-Casanova, Birte Steiniger, Stefanie Schweigle, Holger Clemen, Daniela Zdzieblo, Lisa Starick, Ingrid Müller, Chyung-Ru Wang, Sara Rhost, Susanna Cardell, Elwira Pyz, Thomas Herrmann

**Affiliations:** 1 Institute for Virology and Immunobiology, Julius-Maximilians-University of Würzburg, Würzburg, Germany; 2 Institute of Anatomy and Cell Biology, Philipps-University of Marburg, Marburg, Germany; 3 Department of Microbiology and Immunology, Northwestern University Feinberg School of Medicine, Chicago, Illinois, United States of America; 4 Department of Microbiology and Immunology, Institute of Biomedicine, University of Goteborg, Goteborg, Sweden; New York University, United States of America

## Abstract

**Background:**

CD1d is a nonpolymorphic MHC class I-like molecule which presents nonpeptide ligands, e.g. glycolipids, to NKT cells. These cells are known to have multiple effects on innate and adaptive immune responses and on the development of pathological conditions. In order to analyze CD1d expression and function in the rat, the first rat CD1d-specific monoclonal antibodies (mAbs) were generated.

**Methodology/Principal Findings:**

Two mAbs, WTH-1 and WTH-2, were generated which bound equally well to cell surface-expressed rat and mouse CD1d. Their non-overlapping epitopes were mapped to the CD1d heavy chain. Flow cytometry and immunohistological analyses revealed a nearly identical degree and pattern of CD1d expression for hematopoieitic cells of both species. Notable is also the detection of CD1d protein in mouse and rat Paneth cells as well as the extremely high CD1d expression in acinar exocrine cells of the rat pancreas and the expression of CD4 on rat marginal zone B cells. Both mAbs blocked α-galactosylceramide recognition by primary rat and mouse NKT cells. Interestingly, the two mAbs differed in their impact on the activation of various autoreactive T cell hybridomas, including the XV19.2 hybridoma whose activation was enhanced by the WTH-1 mAb.

**Conclusions/Significance:**

The two novel monoclonal antibodies described in this study, allowed the analysis of CD1d expression and CD1d-restricted T cell responses in the rat for the first time. Moreover, they provided new insights into mechanisms of CD1d-restricted antigen recognition. While CD1d expression by hematopoietic cells of mice and rats was extremely similar, CD1d protein was detected at not yet described sites of non-lymphatic tissues such as the rat exocrine pancreas and Paneth cells. The latter is of special relevance given the recently reported defects of Paneth cells in CD1d^−/−^ mice, which resulted in an altered composition of the gut flora.

## Introduction

CD1 molecules are glycoproteins, which are non-covalently associated with β2-microglobulin and possess an antigen binding groove formed by the α1 and α2 domains. Despite these structural similarities with antigen presenting MHC class I molecules, they considerably differ in other aspects [1], [2], [3]: i) CD1d molecules are rather non-polymorphic whereas classical MHC class I molecules are highly polymorphic, ii) CD1d proteins bind and present antigens containing a lipid or other hydrophobic moieties while MHC class I molecules accommodate and present peptides, iii) so far, CD1 genes have only been identified in mammals and chicken, while MHC class I genes are present in all jawed vertebrates and iv) whereas classical MHC class I molecules are rather similar to each other with respect to function and expression, CD1 genes and molecules differ remarkably between each other and between species in number, expression pattern, type of presented antigens and mode of antigen loading. In humans, the CD1 gene family is composed of five members (*CD1A*, -*B*, -*C*, -*D* and -*E*) which are subdivided into two groups based on the amino acid sequence similarity of the α1 and α2 domains of the encoded proteins [4]. CD1a, -b, and -c belong to group 1; CD1d is the only member of group 2 and CD1e cannot clearly be assigned to either group. In contrast, mice and rats possess only representatives of group 2: mice have two CD1d orthologues (CD1d1 and CD1d2) [5] and rats have one [3], [6], [7]. During the past ten years, the role of CD1 proteins, except for CD1e, as molecules presenting lipid antigens to T cells has been well established [1], [2], [3], [8].

CD1d-restricted T cells often, but not always, express receptors shared with natural killer (NK) cells and are therefore named NKT cells [9]. These cells recognize ligands of endogenous and microbial origins and after activation they secrete very rapidly a wide range of cytokines. NKT cells play an important role in antimicrobial responses, antitumor immunity and the regulation of the balance between tolerance and immunity [10], [11]. NKT cells are divided into type I and II depending on the genes used for the generation of the T cell receptor (TCR) α chain and the reactivity of the TCR to α-galactosylceramide (α-GalCer) which is the first lipid identified to be presented by CD1d. In mice, type I NKT cells (also designated as invariant NKT cells (iNKT cells) or Vα14 NKT cells) express an invariant TCR α chain characterized by AV14-AJ18 rearrangements which pair only with certain β chains resulting in an also limited Vβ repertoire (BV8S2, BV7 and BV2). In the rat, the corresponding α chains are generated by rearrangement of one of its multiple AV14 genes with AJ18. Pairing of these α chains with BV8 containing β chains form α-GalCer reactive TCRs [10], [12], [13]. The type II category includes all CD1d-restricted T cells which do not express the canonical α chain. Although the specificities of this diverse group remain largely unclear, reactivity to endogenous sulfatides has been demonstrated [14], [15], [16], [17].

Of the two CD1d genes present in mice, CD1d2 is only expressed on thymocytes and is of limited functionality with respect to antigen presentation and NKT cell selection [18]. Notably, in C57BL/6 mice a frame shift mutation prevents CD1d2 surface expression [19]. The functional orthologue is CD1d1, which is constitutively expressed on hematopoietic cells although surface expression levels vary among different cell types: Dendritic cells, macrophages and marginal zone (MZ) B cells are the cells with highest levels, followed by B cells and T cells, respectively [19], [20], [21]. Expression in non-lymphatic organs such as liver and lung has also been reported [5], [20], [22]. However, in other tissues like the intestine, the precise localization of CD1d molecules is still a matter of debate [1]. In spite of this, a recent study by Blumberg and colleagues has demonstrated the importance of CD1d expression for gut function since pathogenic and non-pathogenic bacterial intestinal colonization of CD1d deficient mice was increased in comparison to wild type mice [23]. Paneth cells in which CD1d mRNA has been detected by *in situ* hybridization [24] play a crucial role in controlling intestinal homeostasis. Localized at the bottom of the crypts of Lieberkühn, these specialized cells control the microbiota content by secreting antimicrobial peptides (defensins) into the intestinal lumen. Interestingly, Blumberg and colleagues also showed that in CD1d knockout mice, compared to wild type mice, the morphology and content of the secretory granules of the Paneth cells were altered, and more importantly, that degranulation of these cells was defective. Moreover, in wild type mice, degranulation of Paneth cells could be triggered *in vivo* after injection of α-GalCer and *in vitro* after stimulation with α-GalCer and type I NKT cells [23].

Rats serve to investigate numerous biological functions and pathological conditions including models for autoimmune diseases for which a role for CD1d-restricted T cells has been proposed based on studies in mice. Nevertheless, analysis of such cells in the rat has been strongly hampered due to the lack of suitable reagents [13]. The actual knowledge about rat CD1d expression is based on experiments using reverse transcription-polymerase chain reaction (RT-PCR), *in situ* hybridization or polyclonal antiserum. These studies found CD1d to be widely distributed within and outside the hematopoietic system and, as in mice, high levels of CD1d mRNA were detected in Paneth cells [6], [25]. In two additional studies mAbs originally generated against mouse CD1d have been reported to cross-react with rat CD1d. In the first study, the rat IgMs 1H1 and 3C11 [26] were shown to bind a CD1d-like molecule which was detected in the liver but not in the thymus [27]. In the second study, reactivity of mAb 3H3 with rat thymocytes and splenocytes was reported but not further investigated [28]. Hence, prior to our study, appropriate monoclonal antibodies for the analysis of CD1d expression and function in this species were still missing.

Here we report the generation of two monoclonal antibodies with high affinity and similar binding capacities to both, rat CD1d and mouse CD1d1. Both antibodies recognize two distinct epitopes on the CD1d heavy chain and interfere with antigen recognition by CD1d restricted T cells. Apart from this direct demonstration of rat CD1d function and its physiochemical properties, these antibodies allowed us to directly compare CD1d expression of both species revealing only small differences in CD1d expression on corresponding hematopoietic cell subsets. Outside the hematopoietic system we detected high levels of CD1d protein in rodent Paneth cells and exocrine cells of the rat pancreas. The possible implications of CD1d expression by these cells as well as species-specific features of CD1d expression are discussed.

## Results

### Production of two monoclonal antibodies with similar binding capacities to rat CD1d and mouse CD1d1

**Figure 1:**
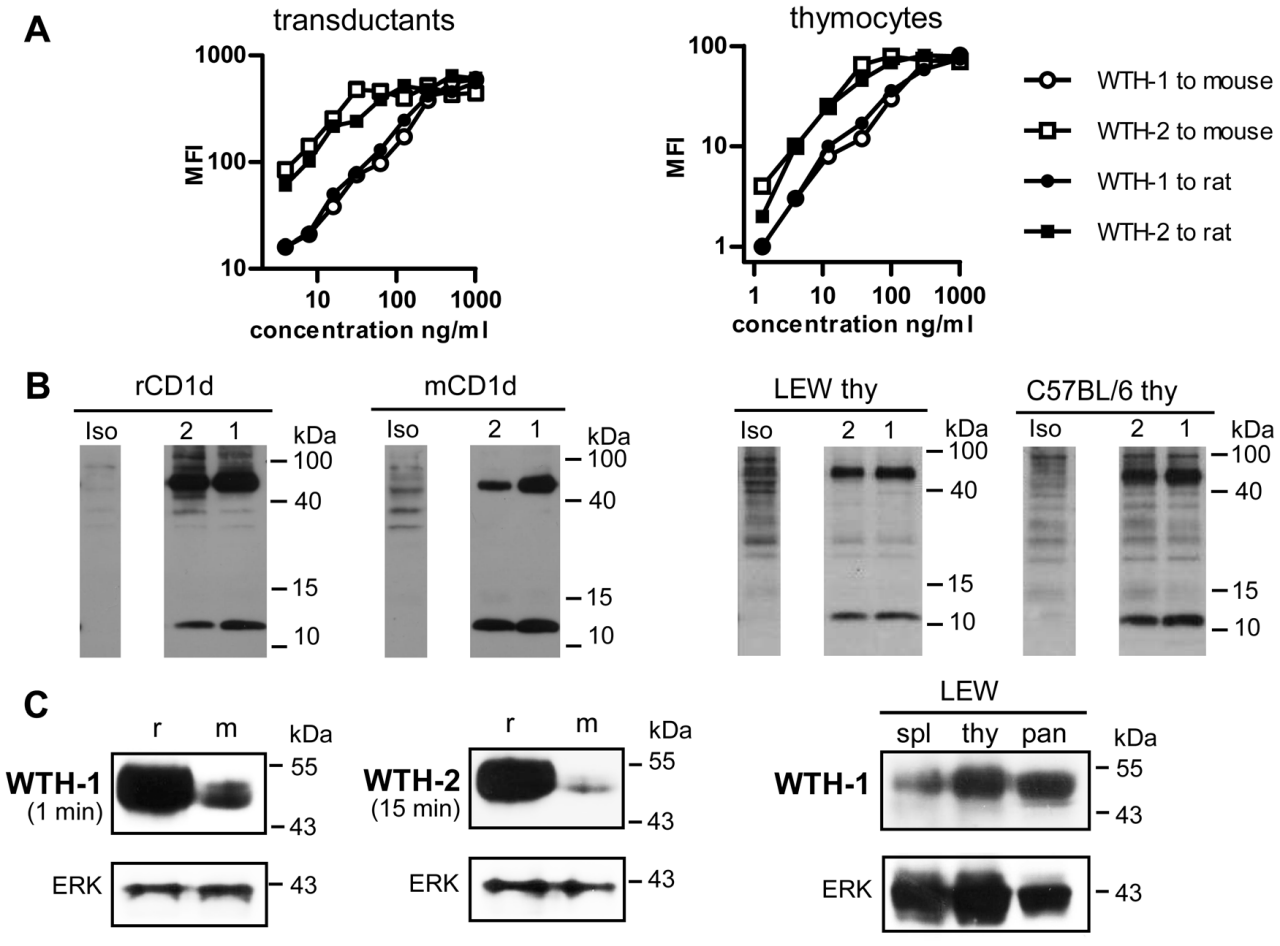
Characterization of two novel anti-CD1d monoclonal antibodies. (A) Titration of WTH-1 and WTH-2 mAbs on rat CD1d or mouse CD1d1 Raji transductants (left) and on C57BL/6 or LEW thymocytes (right). Cells were stained with the indicated concentrations of CD1d-specific antibodies (X axis). After washing, antibodies were detected with PE-labeled donkey anti-mouse IgG and analyzed by flow cytometry. On the Y axis, the geometric mean fluorescence intensity (MFI) is shown for the different antibody concentrations. (B) Immunoprecipitation of biotinylated surface proteins with WTH-1 (1), WTH-2 (2) or isotype control antibodies. Immunoprecipitated material was size separated under reducing conditions on a 15% SDS-PAGE, blotted onto a membrane and detected by addition of streptavidin-HRP. (C) Western blot analysis of proteins derived from rat CD1d (r) or mouse CD1d1 (m) transduced cells - left blots - and from rat tissues: spleen (spl), thymus (thy) and pancreas (pan) - right blots -. Proteins were separated on a 10% polyacrylamide gel under non-reducing conditions and blotted to a membrane. CD1d was detected with the WTH-1 or WTH-2 mAbs. The duration of film exposure for the CD1d immunoblots is indicated under the mAb names. After CD1d detection, the blots were stripped and re-probed with a polyclonal ERK2-specific antibody as protein loading control (lower blots).

In order to produce rat CD1d-specific monoclonal antibodies, CD1d^−/−^ BALB/c mice were immunized with rat CD1d-transduced M12.4.1.C3 cells. Splenocytes of immunized mice were fused with Sp2/0 cells and hybridoma supernatants were screened for reactivity with CD1d by fluorescence activated cell sorting (FACS) analysis of rat CD1d-transduced and untransduced human or mouse cell lines. Two mAbs produced by the hybridomas WTH-1 and WTH-2 were purified and further characterized. To test and compare the binding properties of these antibodies to rat CD1d and mouse CD1d1, transduced cell lines and primary thymocytes (from LEW rats and from C57BL/6 mice) were stained with increasing concentrations of the mAbs and were analyzed by flow cytometry. Both antibodies bind with similar efficacy to mouse or rat CD1d-transduced Raji cells as well as to mouse or rat thymocytes (Fig. 1A). Depending on the antibody preparation, half maximal binding of mAb WTH-2 was observed between 0.2 and 0.5 nM. For the mAb WTH-1 three to five times more antibody was necessary to achieve half maximal binding (Fig. 1A). Not shown is the lack of binding to untransduced Raji cells and thymocytes of CD1d^−/−^ mice. Cross-competition experiments demonstrated that these mAbs recognize different non-overlapping epitopes on CD1d (Table 1).

**Table 1:** WTH-1 and WTH-2 mAbs recognize distinct epitopes.

Cell type	mCD1d1 transductants						LEW rat thymocytes			
Detection mAb	1B1		WTH-1		WTH-2		WTH-1		WTH-2	
Experiment	1	2	1	2	1	2	1	2	1	2
Blocking mAb WTH-1	0	0	100	99	0	0	100	93	7	7
Blocking mAb WTH-2	3	9	7	6	100	100	0	7	100	96

Numbers indicate percentages of binding-inhibition of biotinylated WTH-1 and WTH-2 antibodies by unconjugated blocking antibodies used as “cold” competitors. Biotinylated antibodies were visualized with SA-PE. Inhibition of binding was investigated with mouse CD1d1 transduced Raji cells or LEW thymocytes. Two independent experiments (1 and 2) are shown.

CD1d specificity of the antibodies was further confirmed by immunoprecipitation of biotinylated surface proteins derived from CD1d transductants and primary cells (Fig. 1B). Sodium dodecyl sulfate polyacrylamide gel electrophoresis (SDS-PAGE) analysis of precipitates under reducing conditions revealed two bands with molecular weights similar to those reported for mouse CD1d1 (49–58 kDa) and β2-microglobulin (about 12 kDa) [21]. In SDS-PAGEs using lower acrylamide concentrations (10%) and other set of commercially available molecular weight markers, the precipitated rat CD1d heavy chain appeared with a molecular weight of 50–55 kDa under reducing conditions (data not shown). Moreover, rat CD1d and mouse CD1d1 expressed by human lymphoma cells (Raji) as transgenes had the same molecular size as CD1d expressed by rat and mouse thymocytes. The antibodies were also tested in Western blot analysis. Separation of whole cell lysates under reducing conditions severely impaired the binding of the antibodies (data not shown) but under non-reducing conditions a broad band of about 45–50 kDa appeared, indicating abundant glycosylation (Fig. 1C). These results demonstrate a direct binding of the mAbs to the mature CD1d heavy chain, independent of β2-microglobulin. The mobility of the CD1d heavy chain under non-reducing conditions in SDS-PAGE is also consistent with previous reports on mouse CD1d [26]. Interestingly, in contrast to cell surface staining, both antibodies bound rat CD1d much better than mouse CD1d1 in immunoblots of protein extracts derived from transduced cells which express similar CD1d levels by criteria of cell surface staining and intensity of the EGFP reporter (Fig. 1A and 6B). Moreover, and also in contrast to surface staining, WTH-1 was much more efficient than WTH-2 in Western blot analyses. The right part of Fig. 1C depicts an immunoblot of protein extracts from rat spleen, thymus and pancreas, which demonstrates CD1d expression in these organs. Densitometric evaluation of the films revealed the following ratios between CD1d and ERK2 signals: Spleen, 0.8; thymus, 1.02 and pancreas, 1.72. In SDS-PAGE, pancreatic CD1d had a slightly higher mobility than that from lymphatic tissues. CD1d expression in the pancreas was further analyzed and is described in more detail in a later section of the results.

### CD1d expression on rat and mouse hematopoietic cells

**Figure 2:**
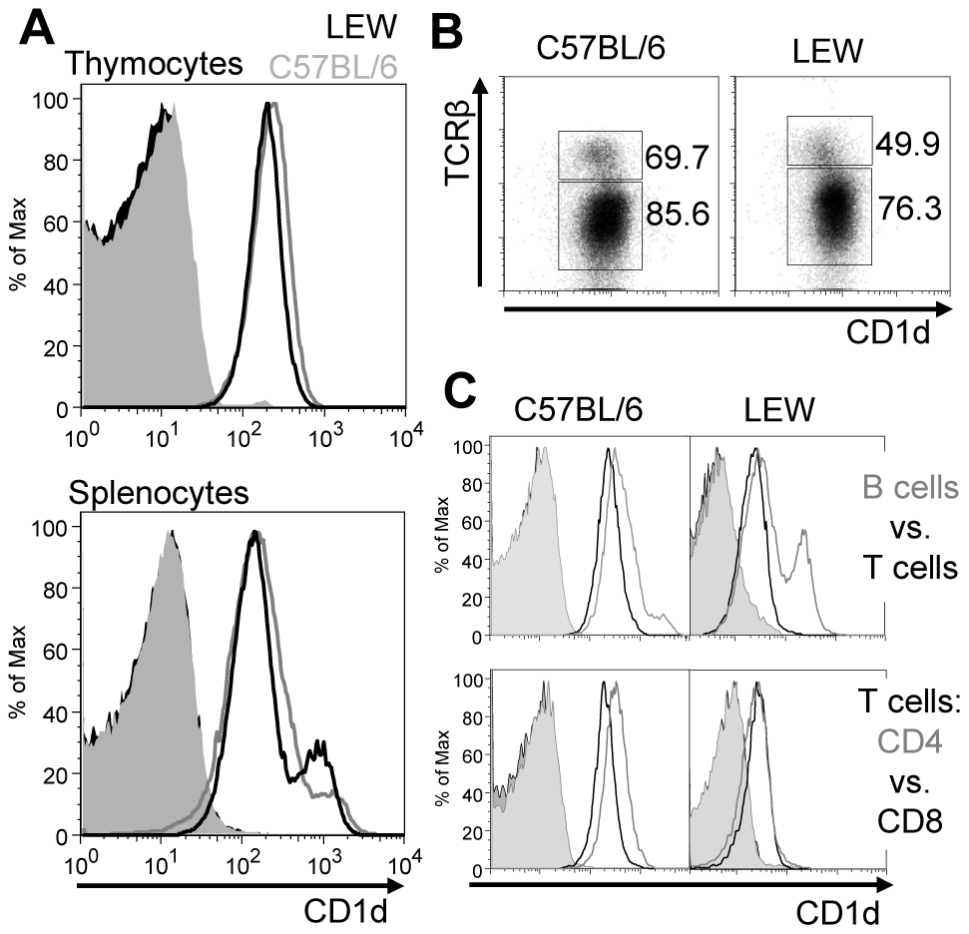
Comparison of CD1d expression levels by rat and mouse primary cells. Representative data from one out of a total of three experiments are shown. (A) Staining of total thymocytes or splenocytes with biotinylated WTH-2 or isotype control antibodies and SA-PE. Gray and black lines correspond to C57BL/6 and LEW cells, respectively. Filled histograms are control stainings. (B) Co-expression of CD1d and TCR on thymocytes was analyzed by two-color flow cytometry. CD1d was stained using the WTH-2 mAb and PE-labeled donkey anti-mouse IgG. For staining of mouse and rat TCRs, H57-597-APCy and R73-bio + SA-APCy were used respectively. Numbers indicate the MFI of anti-CD1d mAb for the gated populations. (C) CD1d expression by B and T cells in the spleen. In C57BL/6 mice, CD1d was analyzed with the biotinylated WTH-2 mAb followed by SA-PE and in LEW rats, since biotinylated mAbs were used for B and T cell identification, CD1d was stained with unconjugated WTH-2 mAb followed by PE-labeled donkey anti-mouse IgG. Filled histograms are control stainings carried out as WTH-2 stainings but with an isotype control antibody. (Upper row) Relative CD1d expression by B and T cells. Histograms show separate multicolor experiments with same overall CD1d staining intensity (Fig. S2), since both, T and B cells, were identified using APCy visualized antibodies in order to avoid unspecific signal due to fluorescence spectral overlap into the PE channel. The gating strategy and the antibodies used are explained in detail in figure S2. Gray and black lines correspond to B and T cells, respectively. (Lower row) Histograms show CD1d expression by CD4 and CD8 positive T cells. The gating strategy and used antibodies are explained in detail in the figure S3. Gray and black lines represent CD4^+^ and CD8^+^ T cells, respectively.

The WTH-1 and WTH-2 mAbs allowed us to directly compare CD1d surface expression on mouse and rat hematopoietic cells by flow cytometry. The results obtained with the two mAbs were nearly the same, therefore, only stainings performed with the WTH-2 mAb are shown. At first thymocytes and splenocytes of five different inbred rat strains which represent two different CD1d alleles were analyzed; F344 versus LEW, BN, PVG and DA. This analysis revealed no allele specific differences in cell surface CD1d expression levels but inconsistencies between our data and published results on CD1d sequences and strain specificity of these alleles, which are described in greater detail in the supplementary material (Table S1 and Fig. S1). Comparison of CD1d expression in LEW rats and C57BL/6 mice showed very similar staining intensities on thymocytes and most splenocytes (Fig. 2A). The only striking difference was the higher proportion of CD1d high cells among rat splenocytes which, as shown later, can be attributed to MZ B cells [21]. This is consistent with the very broad marginal zone and large number of these cells found in rats [29], [30], [31].

Co-staining of CD1d and TCR in thymocytes revealed that in rats, as in mice [19], TCR low cells, which are double positive thymocytes, express higher levels of CD1d than TCR high cells, which are single positive thymocytes (Fig. 2B). CD1d expression by mouse T and B cells of the spleen and lymph nodes has been extensively studied [19], [20], [21], [32]. CD1d surface expression is lower on T cells compared to B cells and among T cells, is higher on CD4 T cells than on CD8 T cells. Using the WTH-1 and WTH-2 antibodies we reproduced the findings obtained in mice, and observed that rat B cells also express higher CD1d levels than T cells (Fig. 2C and Fig. S2). However, in rats the pattern of CD1d distribution among T cell subpopulations is different than in mice. CD1d levels on rat CD4 T cells are not higher than those on CD8 T cells of the rat, as demonstrated by different staining and gating strategies (Fig. 2C and Fig. S3).

**Figure 3:**
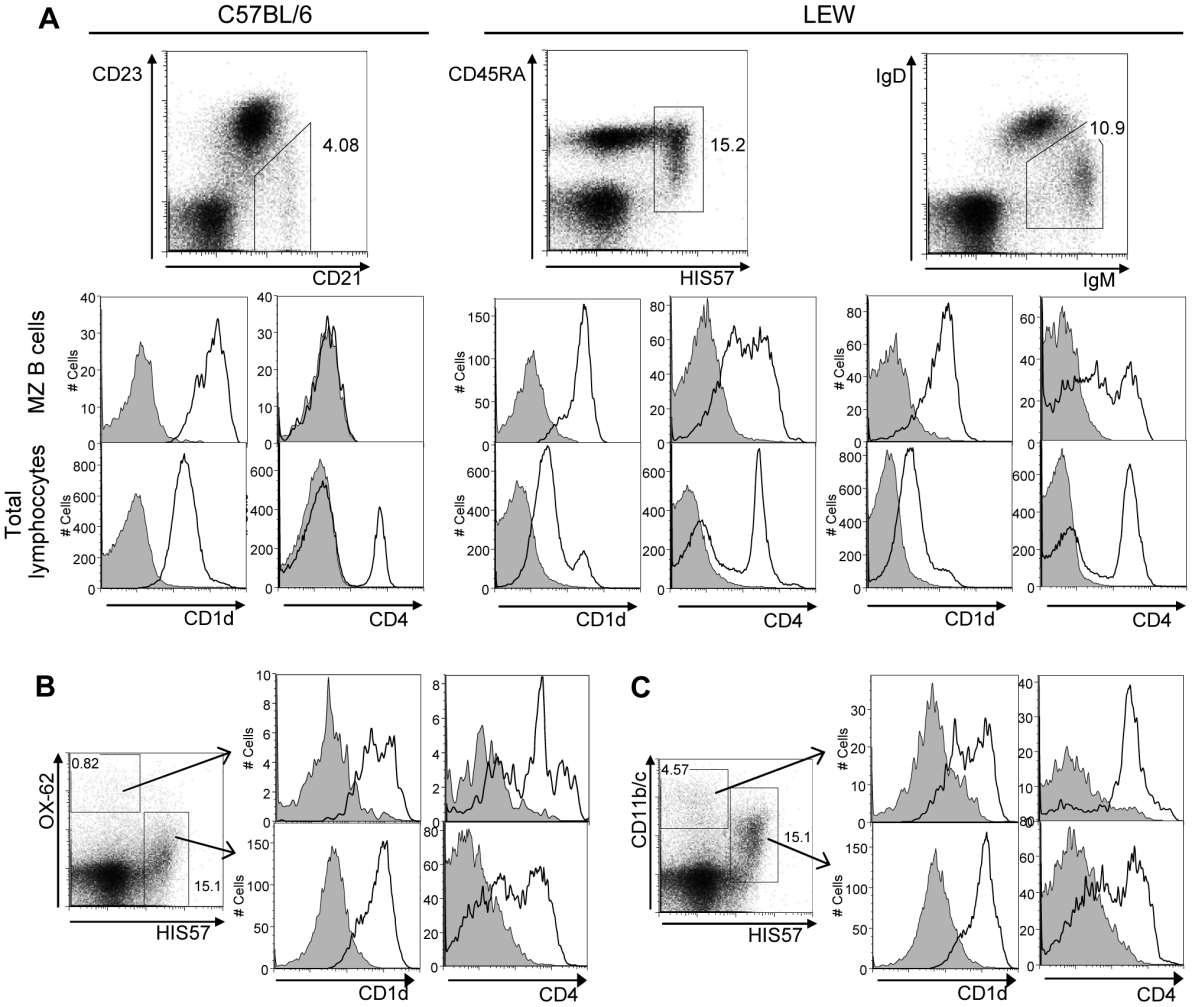
CD1d and CD4 expression by MZ B cells, dendritic cells and macrophages from the spleen. One representative of three independent experiments is shown. Dot plots illustrate gating strategies. Numbers indicate the percentages of gated cells. Histograms show CD1d and CD4 expression levels. (A) MZ B cell analysis. In C57BL/6 mice, CD1d was stained with biotinylated WTH-2 mAb + SA-Cy5-PE and CD4 with RM4-APCy. In LEW, CD1d was detected with unconjugated WTH-2 mAb followed by PE-labeled donkey anti-mouse IgG antibody and CD4 with OX-35 labeled with PE-Cy5 unless otherwise indicated. Upper row histograms show gated MZ B cells, whereas, lower row histograms show total lymphocytes. MZ B cells in C57BL/6 mice were identified by gating on CD21 hi (7G6-FITC) and CD23 low/negative (B3B4-PE) cells. In LEW rats, MZ B cells were stained with two different marker combinations: CD45RA (OX-33-FITC)/HIS57-biotin + SA-APCy and IgM (G53-238-FITC)/IgD (MARD-3-biotin + SA-APCy), respectively. Analysis of CD1d and CD4 in MZ B cells defined as HIS57 and CD45RA positive cells was carried out with one single multicolor experiment. Expression of CD1d and CD4 in MZ B cells defined as IgD low and IgM high cells was determined in separated multicolor experiments as both, the CD1d and the CD4 specific antibodies, were visualized with the PE fluorochrome (CD1d: WTH-2 + PE-donkey anti-mouse IgG, CD4: OX-35-PE). (B) CD1d and CD4 expression by LEW dendritic cells (OX-62 + PE donkey anti-mouse IgG secondary antibody) and MZ B cells (HIS57-biotin and SA-APCy). CD1d was detected with WTH-2-FITC and CD4 with OX-35-PE-Cy5. (C) CD1d and CD4 expression by LEW macrophages - defined as CD11b/c^+^ cells (OX-42-PE) - and MZ B cells (HIS57-biotin + SA-APCy). CD1d was stained with unconjugated WTH-2 followed by FITC-labeled donkey anti-mouse IgG. For CD4 detection, OX-35-PE-Cy5 antibody was used.

In the mouse, in addition to MZ B cells, other antigen presenting cells [21] also show very high CD1d levels. Analysis of mouse MZ B cells (CD21high/CD23low) with the mAbs reported here confirmed their high CD1d expression levels (Fig. 3A). Rat MZ B cells defined either by combinations of the B cell marker CD45RA (OX-33) and the MZ B marker HIS57 or by gating on IgM high/IgD low cells [29] are CD1d high as well (Fig. 3A). Surprisingly, most MZ B also expressed CD4 and nearly half of them as much as other splenocytes (e.g. CD4 T cells) (Fig. 3A). These findings were confirmed with a second CD4 specific mAb (OX-38) and in another inbred rat strain (F344) (data not shown). Mouse MZ B cells were CD4 negative (Fig. 3A). CD1d expression on rat dendritic cells (OX-62^+^) and macrophages (CD11b/c^+^) was also analyzed and compared to that of MZ B cells, identified as HIS57^+^ cells in this case. The stainings depicted in figures 3B and C show that, similar to mouse antigen presenting cells, CD1d levels on rat dendritic cells and macrophages are as high as those of MZ B cells. Moreover, these stainings exclude that the CD4^+^ cells observed among the HIS57^+^ gated cells are macrophages or dendritic cells. Last, we also analyzed CD1d expression on natural killer cells from the spleen which were defined as NK1.1 or NKR-P1A positive cells in mice and rats, respectively. In C57BL/6 mice, NK1.1 positive cells express lower CD1d levels compared to the rest of splenocytes, whereas in rats NKR-P1A positive cells and T cells express CD1d at similar levels (data not shown).

### Immunohistological analysis of CD1d expression in lymphatic organs

**Figure 4:**
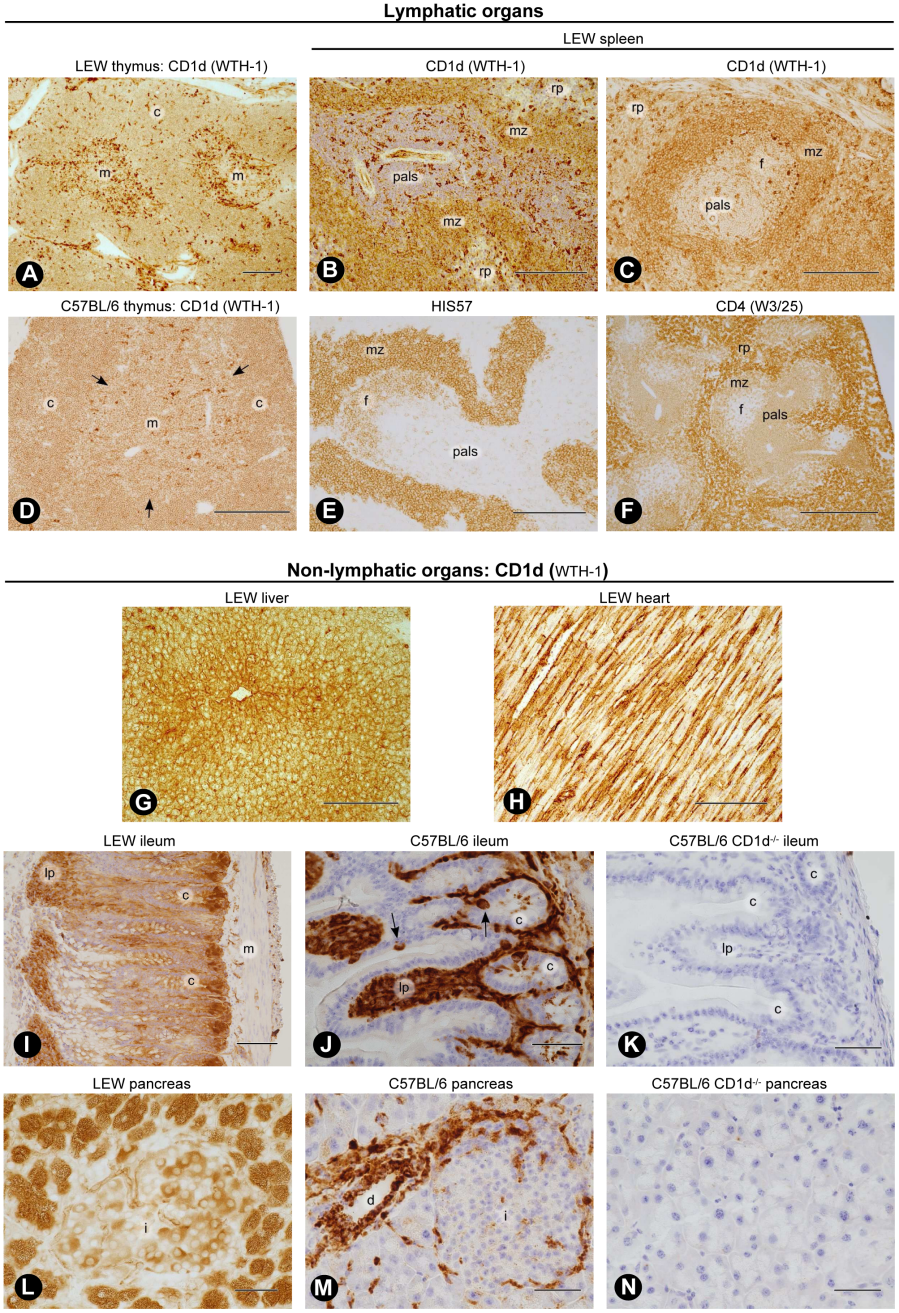
CD1d distribution in lymphatic and non-lymphatic organs. ABC technique with unconjugated mAb WTH-1 unless otherwise stated. (A) LEW thymus. Bar  = 500 µm. (B) Longitudinal section of LEW spleen. Hemalum counterstain. Bar  = 250 µm. (C) Cross-section of LEW splenic white pulp. WTH-1 biotin-conjugated primary antibody. Bar  = 250 µm. (D) C57BL/6 mouse thymus. Arrows indicate cortico-medullary boundary. Biotin-conjugated antibody. Bar  = 250 µm. (E) HIS57 staining in LEW splenic white pulp. Bar  = 250 µm. (F) CD4 (mAb W3/25) expression in LEW spleen. Bar  = 500 µm. (G) LEW liver. Bar  = 250 µm. (H) LEW heart. Hemalum counterstain. Bar  = 250 µm. (I) LEW ileum. The epithelium at the tips of the villi (left rim of picture) is not preserved. Biotinylated WTH-1 and hemalum counterstain. Bar  = 100 µm. (J) C57BL/6 ileum. Arrows indicate CD1d positive enteroendocrine cells in the epithelium. Biotin-conjugated primary antibody, tyramide-amplified ABC technique and hemalum counterstain. Bar  = 50 µm. (K) C57BL/6 CD1d^−/−^ ileum. Biotin-conjugated primary antibody, tyramide-amplified ABC technique and hemalum counterstain. Bar  = 50 µm. (L) LEW pancreas. Biotinylated WTH-1. Bar  = 50 µm. (M) C57BL/6 pancreas. Biotin-conjugated primary antibody, tyramide-amplified ABC technique. and hemalum counterstain. Bar  = 50 µm. (N) C57BL/6 CD1d^−/−^ pancreas. Biotin-conjugated primary antibody, tyramide-amplified ABC technique and hemalum counterstain. Bar  = 50 µm. Abbreviations in lymphatic organs: m, medulla; c, cortex; rp, red pulp; pals, periarteriolar lymphatic sheath; mz, marginal zone and f, follicle. Abbreviations in non-lymphatic organs: c, crypts; lp, lamina propria; m, smooth muscle cells of the gut wall; i, islet of Langerhans and d, interlobular duct.

CD1d distribution was analyzed in rat and mouse thymus and spleen by immunohistology with mAbs WTH-1 and WTH-2. In rats, results obtained with both antibodies were almost identical except for certain cell types such as endothelia in large vessels which were less efficiently stained by mAb WTH-2. Thus, staining results are only shown for mAb WTH-1 (Fig. 4). The histological expression pattern of CD1d in both organs was identical in rats and mice, but mice exhibited overall a lower staining intensity.

In both species monocytes, certain macrophages and dendritic cells were the most strongly reactive cells in the organs investigated (Fig. 4A–D). Reactivity of endothelial cells was variable. Macrophages and dendritic cells were especially abundant in the thymic medulla of 12-week-old rats and were less conspicuous in 8- to 10-week-old mice (Fig. 4A, D). Cortical and medullary thymocytes were clearly positive in both species and there were only minimal differences in staining intensity among both cell types (Fig. 4A, D).

In rat spleens a graded intensity of CD1d expression was evident in white pulp lymphocytes, which corresponded to the data obtained by flow cytometry (Fig. 4 B, C). MZ B cells were most strongly stained, follicular B cells exhibited intermediate staining and T cells in the periarteriolar lymphatic sheath (PALS) were the least reactive cells. Marginal metallophilic macrophages surrounding the follicles and dendritic cells in the PALS were the most conspicuous cells in the rat white pulp (Fig. 4B, C). In the splenic red pulp, monocytes and granulocytes were the most strongly stained cells. The red pulp also contained a large number of cells with intermediate CD1d levels whose identity remains to be identified.

Flow cytometric studies had revealed that rat MZ B cells express CD4 (Fig. 3). Therefore, we also analyzed the presence of CD4 and the HIS57 antigen in the spleen by immunohistology and confirmed that mAb HIS57 predominantly stained MZ B cells (Fig. 4E). A subpopulation of follicular B cells in a superficial cap-like accumulation and dendritic-like cells in the PALS were also positive, but their staining intensity was low. Consistent with flow cytometric data using anti-CD4 mAbs OX-35 and OX-38, immunohistology with mAb W3/25 clearly visualized that CD4 was not only expressed by a large number of T lymphocytes in the PALS and by typical monocytes and macrophages in the red pulp, but also by MZ B cells. Follicular B cells, however, remained unstained (Fig. 4F).

### CD1d expression in non-lymphatic organs

Using mAbs WTH-1 and WTH-2 the *in situ* expression of CD1d was also investigated in the liver, heart, small intestine and pancreas of rats. Since CD1d was detected in unexpected sites in the rat pancreas and small intestine, these organs were also analyzed in the mouse. In the rat, both antibodies produced very similar staining patterns in each organ with an intensity comparable to that found in lymphatic organs.

CD1d was widely expressed by rat non-lymphatic parenchymal cells such as hepatocytes, cardiomyocytes and pancreatic acinar cells (Table S2, Fig. 4 G–I, L). Moreover, capillary and large vessel endothelia, interstitial macrophages and dendritic cells were strongly positive in all organs investigated. Due to the intense endothelial staining it was however difficult to identify cells directly adjacent to microvessels such as von Kupffer cells and stellate cells in the liver (Fig. 4G). Capillary endothelial reactivity also precluded unequivocal recognition of intercalated duct epithelia in the pancreas. Likewise, enteroendocrine cells could not be distinguished among the mass of CD1d^+^ enterocytes (Fig. 4I). In contrast, smooth muscle cells expressed CD1d at the detection limit.

Two peculiarities deserve a detailed comment. First, in the rat ileum Paneth cells, which were easily identified due to their large apical granules and their location at the bottom of the crypts, were the most strongly stained epithelial cells. The strong staining reaction appeared to be predominantly associated with the exocrine granule membranes (Fig. 4I). Second, rat pancreatic acinar cells also exhibited massive staining of apical exocrine granules. This phenomenon was still visible at mAb WTH-1 dilutions of more than 1∶100.000 (Fig. 4L) and it corresponded to the results of the Western blot analyses (Fig. 1C). CD1d expression in rat endocrine islet cells was variable. Some, but not all, of the endocrine cells located more centrally in the islet (most likely insulin-producing beta cells) were strongly stained while the peripheral rim of glucagon-producing alpha cells were hardly reactive (Fig. 4L).

When comparing the staining patterns observed in rats with those in mice, mAbs WTH-1 and WTH-2 gave inferior staining in mouse than in rat organs. This discrepancy was especially evident in non-lymphatic organs. In addition, mAb WTH-2 produced a much lower signal in mice than mAb WTH-1. Thus, mAb WTH-1 was preferentially used. In order not to miss low-grade expression, mouse non-lymphatic organs were stained using a highly sensitive tyramide amplification procedure with inclusion of C57BL/6 CD1d^−/−^ mouse organs as negative controls (Fig. 4J, K, M, N). Mouse non-lymphatic organs exhibited a much reduced reactivity for CD1d in parenchymal cells and large vessel endothelia, while expression in macrophages, dendritic cells, putative fibroblasts and certain epithelial cells did not differ from that in the respective rat cells (Table S3 and Fig. 4I, J, L, M). In the ileum of mice, enterocytes appeared negative, while enteroendocrine cells, the apical granules (or a subpopulation of them) in Paneth cells and the villous stroma were unequivocally positive (Fig. 4J). In contrast to rat pancreas, only few cells in mouse exocrine pancreatic acini contained weakly CD1d^+^ granules (Fig. 4M). The strongest expression of CD1d in mouse pancreas occurred in the epithelium of larger ducts and in elongated interacinar cells which might correspond either to intercalated duct epithelia or to capillary endothelia (Fig. 4M). Endocrine islet cells were negative to faintly positive (Fig. 4M). Identical results were obtained in mouse strains, which express CD1d2, such as BALB/c or CBA/N (data not shown).

In summary, in non-lymphoid organs CD1d expression varies considerably between different cell types and between rat and mouse. Moreover, our results show CD1d expression by unpredicted cell types as rat exocrine pancreatic cells.

### Effects of WTH-1 and WTH-2 mAbs on CD1d antigen presentation to type I and II NKT cells

**Figure 5:**
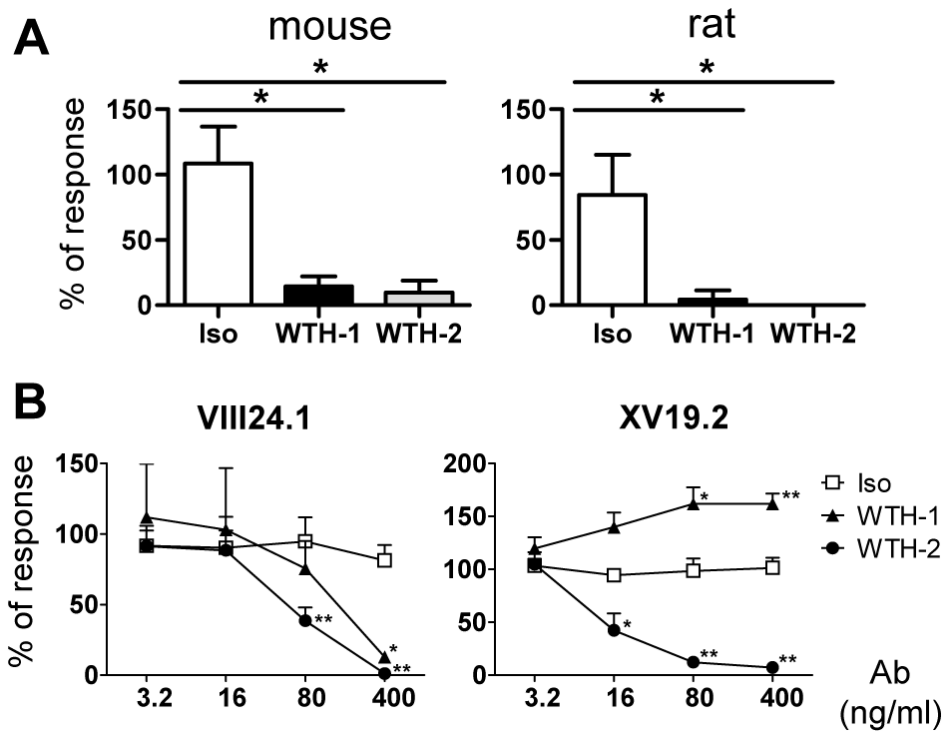
Effects of anti-CD1d mAbs on antigen presentation to type I and II NKT cells. (A) Blocking of type I NKT cells responses by the mAbs WH1-1 and WTH-2. IL-4 production was analyzed by measuring cytokine release into the culture supernatant 24 hours after initiation of the cultures by ELISA. Total splenocytes from C57BL/6 mice or F344 rats were cultured with α-GalCer (10 ng/ml), in the presence or absence of WTH-1, WTH-2 or isotype control antibodies (with a final saturating concentration of 3.6 µg/ml). Results were normalized to 100% for IL-4 production without antibody. Bars show means + standard deviation (SD) of the results obtained in three independent experiments. IL-4 concentrations without antibody were 9.9, 3.3 and 6.5 pg/ml and 15.8, 28.4 and 20.9 pg/ml for rat and mouse cultures, respectively. (B) Effects of the antibodies on CD1d antigen presentation to type II NKT cells. IL-2 was measured by ELISA in supernatants of co-cultures of type II NKT cell hybridomas and CD1d-transduced cells. Raw data were normalized to IL-2 production without antibody equaling 100%. The graphics represent the mean values + SD obtained from three independent experiments. The left graph shows IL-2 production by 5×10^4^ VIII24.1 T cell hybridoma cells after co-culture with 5×10^4^ mCD1d1-transduced LBB cells. IL-2 concentrations without addition of antibodies, in the three independent experiments, were 575, 415 and 572 pg/ml, respectively. The right graph gives the corresponding results for 1×10^4^ XV19.2 T cell hybridoma cells after co-culture with 5×10^4^ mCD1d1 transduced Raji cells. IL-2 concentrations without added antibody in the three independent experiments were 33.9, 101.6 and 22.6 pg/ml, respectively. To determine differences between cultures where isotype control antibody or anti-CD1d mAbs were added, two-tailed paired t-test was used. * and ** indicate p-values <0.05 and <0.005, respectively.

Upon stimulation with the potent antigen α-GalCer, type I NKT cells secrete Th1 and Th2 cytokines very rapidly, being the only lymphocytes described to date that can secrete large amounts of interleukin (IL) 4 within a few hours after first antigen contact. Therefore, in order to test possible effects of WTH-1 and WTH-2 mAbs on CD1d antigen presentation to rat or mouse type I NKT cells, splenocytes from both species were cultured with α-GalCer in the presence or absence of the anti-CD1d mAbs and after 24 hours, culture supernatants were removed and IL-4 concentrations were determined by enzyme-linked immunosorbent assay (ELISA). As controls, splenocytes were incubated in parallel with β-GalCer, concanavalin A (Con A) or culture medium only (Table S4). As shown in figure 5A, addition of the antibodies blocked IL-4 release in cultures stimulated with α-GalCer, regardless of the species origin. A toxic or unspecific effect of the antibodies could be ruled out as they did not inhibit cytokine production after Con A stimulation and the isotype control did not affect α-GalCer-dependent cytokine release (Table S4 and Fig. 5A).

To investigate the impact of the antibodies on CD1d recognition by type II NKT cells, IL-2 production by two different CD1d-restricted autoreactive T cell hybridomas was analyzed in the presence of WTH-1, WTH-2 or an isotype matched control antibody. The mouse derived VIII24.1 hybridoma which expresses a non-invariant TCR containing the AV3S2 and BV9 gene segments [14] was co-cultured with LBB cells transduced with mouse CD1d1. A dose dependent inhibition of IL-2 production was observed when the anti-CD1d mAbs were added in comparison to the isotype matched control which had no significant effect (Fig. 5B). This demonstrates that both antibodies were able to prevent the presentation of LBB endogenous ligands by CD1d to the VIII24.1 hybridoma. Effects on recognition of endogenous ligands presented by mouse CD1d1-transduced Raji cells to the XV19.2 hybridoma were also tested (Fig. 5B). While WTH-2 efficiently blocked the activation of the XV19.2 cells, WTH-1 increased activation of this hybridoma in a dose dependent manner.

### Epitope mapping of mAbs WTH-1 and WTH-2

**Figure 6:**
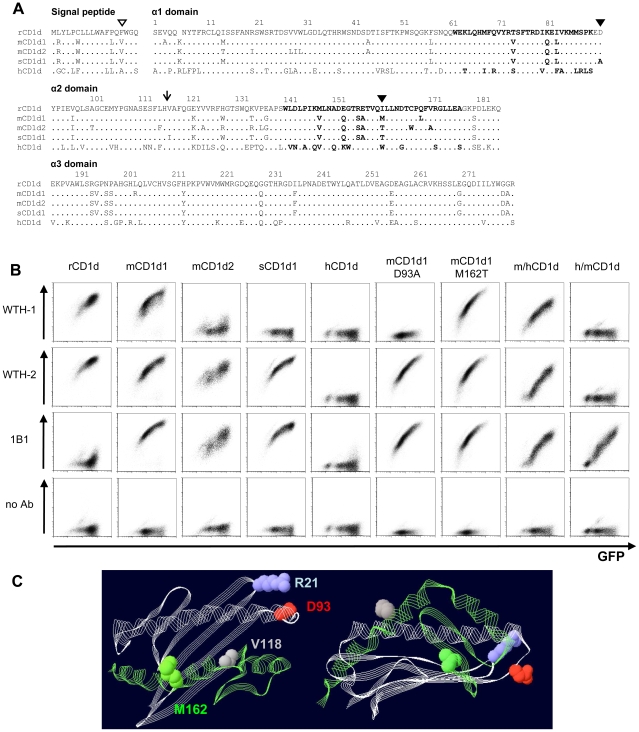
Epitope mapping of CD1d-specific mAbs. (A) Alignment of the amino acid sequences of the extracellular domains of the CD1d molecules used in this study. α-helical regions are illustrated in bold. The open triangle points out the localization of the rat CD1d allelism. Mutations in mouse CD1d1 are highlighted with closed triangles. The arrow indicates where mouse/human chimeras were joined. Numbers show rat CD1d amino acid positions. Accession numbers for the amino acid sequences can be found in GenBank: rCD1d (*Rattus norvegicus* CD1d), BAA82323; mCD1d1 (*Mus musculus* CD1d1), NP_031665; sCD1d1 (*Mus spretus* CD1d1), ACM45455; hCD1d1 (*Homo sapiens* CD1d), NP_001757. The mCD1d2 (*Mus musculus* CD1d2) amino acidic sequence differs in one amino acid in the signal peptide (tryptophan, position −13) from the sequence published under the accession number: P11610. (B) Binding capacity of mAbs to different CD1d molecules. Raji cells were transduced with CD1d molecules using a retroviral expression system. Bicistronic EGFP in the CD1d expression vectors served as reporter gene. Cells were stained with unconjugated WTH-1 or WTH-2 mAbs followed by PE-labeled donkey anti-mouse IgG or PE-labeled 1B1 mAb and were analyzed by flow cytometry. All primary antibodies were used at a final concentration of 250 ng/ml. (C) Localization of relevant amino acids in the CD1d tertiary structure. The model depicts part of the co-crystal of the PBS25 glycolipid and mouse CD1d (PDB: 3GMP) and it was visualized using PDB Swiss Viewer Deep View v4.0 [52] (http://www.expasy.org/spdbv/). The α1 and α2 domains are shown. Gray and green ribbon diagrams highlight the regions constituting the N- and C-terminal parts, respectively, of the chimeric molecules. Spheres visualize the amino acids discussed in the text: aspartic acid (D) 93 in red, methionine (M) 162 in green, valine (V) 118 in gray and arginine (R) 21 in blue.

In order to better understand the different effects of the mAbs on antigen presentation by CD1d, we aimed to identify the epitopes recognized by each antibody. In addition to the two *CD1d* genes present in commonly used inbred mouse strains which are derived from *Mus musculus* or *Mus domesticus*, recently, different CD1d1 sequences from *Mus spretus* and *Mus castaneus* have been reported [33]. Moreover, the human CD1d amino acid sequence is highly conserved when compared to mouse CD1ds and rat CD1d (Fig. 6A). To allow a first screening of possible epitope candidates, some of these CD1d variants were expressed in Raji cells and the antibody binding capacity was assessed by flow cytometry (Fig. 6B). Furthermore, chimeras between mouse CD1d1 and human CD1d (m/h CD1d and h/m CD1d) were produced and were also tested for their reactivity with the antibodies WTH-1 and WTH-2. The arrow in figure 6A points out the position where the regions of mouse CD1d1 and human CD1d were exchanged. The structure models shown in figure 6C illustrate the species origen of the different parts of the chimeric CD1d molecules.

The WTH-1 antibody recognized rat CD1d (rCD1d) and CD1d1 from *Mus musculus* (mCD1d1), whereas it did not bind to the *Mus spretus* CD1d1 variant (sCD1d1) and stained only very weakly *Mus musculus* CD1d2 (mCD1d2) transductants (Fig. 6B). Only three amino acid residues are unique to the sCD1d sequence when compared to rCD1d and mCD1d1: alanine (A), isoleucine (I) and threonine (T) at positions 93, 118 and 162, respectively. The aspartic acid 93 and the methionine 162 which are exposed at the surface of mCD1d1 were exchanged by their sCD1d counterparts: alanine and threonine, and named as: mCD1d1D93A and mCD1d1M162T (Fig. 6). These mutants, as the other CD1d molecules, were expressed in Raji cells and analyzed by flow cytometry (Fig. 6B). The aspartic acid at positions 92 and 93 of rat CD1d and mouse CD1d1, respectively, were identified as part of the WTH-1 epitope because cells expressing mCD1d1D93A were not stained by this mAb. Moreover, staining of the cells transduced with the mouse/human chimeras confirmed these results. WTH-1 mAb bound only very weakly, if at all, to mCD1d2 despite the conserved aspartic acid at position 93 (Fig. 6A and B). There are, in principle, several unique substitutions from which this could result. Among these substitutions, the hydrophobic isoleucine in mCD1d2 which substitutes the positively charged arginine at position 21 of mCD1d1 (blue sphere in Fig. 6C) most likely affects the orientation of the aspartic acid 93 or the direct contact with the antibody.

The WTH-2 antibody detected all murine CD1d molecules tested but did not bind to human CD1d (hCD1d). hCD1d cell surface expression was confirmed with the CD1d42 mAb [34], which also bound to m/hCD1d but not to h/mCD1d chimeras (data not shown). Staining of the chimeras, mapped the epitope recognized by WTH-2 to the α1 domain and the first 26 amino acids of the α2 domain of mCD1d1, since binding to the h/m chimera was lost, but binding to the m/h chimera was conserved. The aspartic acid 93 can be excluded to be part of the WTH-2 epitope since this mAb also stained mCD1d1D93A-transduced cells (Fig. 6B).

Furthermore, we also tested antibody reactivity of the commercially available anti-mouse CD1d mAb 1B1 [20] against all CD1d variants. 1B1 bound all mouse CD1d molecules, stained rCD1d transductants very weakly and did not recognize hCD1d-transduced cells at all. However, there was a moderate binding of 1B1 to both human/mouse chimeras, suggesting that both parts of the mouse molecule contribute to the formation of the epitope (Fig. 6B). These results and the competition experiments make clear that all these mAbs bind to rather distinct parts of the CD1d molecules.

## Discussion

Up to now, the analysis of rat CD1d expression and function was limited by the lack of CD1d-specific monoclonal antibodies. Here we report the production and detailed characterization of two monoclonal antibodies of dual specificity for rat CD1d and for mouse CD1d1. These mAbs are not only valuable tools for the functional and biochemical characterization of CD1d in either species but also allow a side by side comparison of CD1d expression in mice and rats.

At saturating conditions, both mAbs bound mouse CD1d1 and rat CD1d expressed at the cell surface equally well, i.e. irrespectively of whether they had been expressed by a transduced human B cell lymphoma, thymocytes or other lymphoid cells (Fig. 1A, 2 and 6B). In these experiments the avidity of mAb WTH-2 was three to five-fold higher than that of WTH-1. This was in contrast to what was found in Western blot analyses (Fig. 1C), immunohistology (Fig. 4), and sometimes also immunoprecipitation (Monzon-Casanova and Herrmann, unpublished data), where WTH-1 was superior to WTH-2 and where binding to rat CD1d was more efficient than to mouse CD1d1. Altogether, these findings suggest that the WTH-1 epitope is more resistant to denaturation than the WTH-2 epitope and also that denaturation affects more the epitopes recognized in mouse CD1d1 than in rat CD1d. Nonetheless, it remains to be investigated, whether these differential binding efficacies result from protein denaturation and/or effects of detergents or alcohols on CD1d bound lipid-containing antigens.

Both mAbs precipitated CD1d heavy chain and β2-microglobulin from extracts of rat or mouse CD1d transductants as well as from rat or mouse thymocytes. In Western blot analyses, the mobility of the CD1d heavy chain was slightly higher when the extracts were separated by SDS-PAGE under non-reducing as compared to reducing conditions (data not shown), probably as consequence of its more compact structure maintained by the intact intramolecular disulfide bridges. These last results demonstrate the localization of both epitopes on the CD1d heavy chain and, to our knowledge, are also the first demonstration of IgG mAbs binding to mouse or rat CD1d heavy chains in a Western blot.

The competition experiments revealed that the mAbs bind to distinct non-overlapping epitopes. This property allows validation of results e.g. in tissue distribution analysis by using two such antibodies, as it has been done in this study. In addition, the antibodies are highly suitable for the design of a Sandwich ELISA and can be used to detect CD1d in serum or to monitor concentrations of CD1d during preparation and purification of soluble CD1d (Monzon-Casanova and Herrmann, unpublished data). Analysis of mAb reactivity against various CD1d transductants allowed a partial mapping of the epitopes as discussed in the results section, which in case of the mAb WTH-1 is likely to contain the loop linking the α1 and the α2 domains of the CD1d molecule.

CD1d expression levels at the cell surface of different hematopoietic cells in the rat and in the mouse were highly similar. The only notable difference between these species was observed among CD4 and CD8 peripheral T cells. Nonetheless, the difference was rather small and a physiological role for CD1d on mature T cells still has to be described. The nearly identical CD1d expression in thymocytes is of special interest because, apart from mediating the selection of NKT cells [35], different levels of CD1d in this cell type considerably affect NKT cell frequency and state of activation [36].

At a first glance, CD1d expression by dendritic cells and macrophages was also quite similar for both species. It will be interesting to learn whether it can be used as a marker to further differentiate antigen presenting cell subsets, or in the analysis of other cell types such as granulocytes or monocytes. Altogether, it can be expected that CD1d-restricted immune responses show no qualitative differences between mice and rats, and if that was the case, that such differences would not be intrinsic to differences in CD1d expression on hematopoietic cells.

A major surprise was the previously undescribed substantial CD4 expression on rat MZ B cells, which was shown to be specific using three different monoclonal antibodies which bind to two different domains of the CD4 molecule [37]. Retrospectively, it is difficult to explain, why this observation has not been reported before. The functional implications of this finding are unclear, but it is important to know that any experimental setting addressing CD4, e.g. in depletion studies with CD4 specific antibodies in the rat, can be expected to affect not only CD4 T cells and to some extent antigen presenting cells but MZ B cells as well. Since rats and humans are more similar to one another with respect to CD4, CD8 and MHC class II expression by immune cells than rats and mice [38], [39], [40], it may be worthwhile to reinvestigate CD4 expression on human MZ B cells as well.

Rat CD1d is also widely expressed outside the hematopoietic system being found in unexpected sites as the exocrine pancreas. In contrast to lymphatic tissues, big differences were observed between mice and rats in certain non-hematopoietic cells. In the rat pancreas, the vast expression of CD1d by exocrine acinar cells detected by immunohistochemical analyses is not only supported by our Western blot results, but also by two independent proteomic analyses which also identified CD1d in pancreatic exocrine granules [41], [42]. The slightly lower molecular weight of the pancreatic CD1d compared to that from extracts of spleen or thymus, could indicate different processing of CD1d during intracellular maturation e.g. proteolytic cleavage of membrane bound material or generation of a truncated form. In the small intestine, CD1d expression varied considerably between mouse and rat enterocytes, but detection of CD1d was possible in Paneth cells of both species. The biological function of CD1d in acinar pancreatic cells and in Paneth cells in the rat remains to be investigated. Nonetheless, since CD1d is mainly found in exocrine granules and mouse CD1d-deficient Paneth cells presented intrinsic alterations in their degranulation capacity as well as in their granular content and morphology [23], CD1d expressed by exocrine cells might participate in a biological process other than antigen presentation. Indeed, the human CD1e isoform is an example of such a different function because it is cleaved into a soluble form which facilitates glycolipid processing [8], [43].

Interestingly, although a substantial variability has been found between number and types of CD1 isoforms in different clades of placental mammals, complete CD1 deficiency has not been reported yet [3]. This not only supports the idea that CD1 molecules fulfill essential biological functions, but also implies that the function of CD1 isoforms may vary between species and that at least to some extent, CD1 isoforms could functionally substitute each other. In muridae CD1d is the only CD1 isoform. Therefore, one may speculate a need for “multitasking” of CD1d in these species. Such a requirement may explain the – in comparison to humans – less restricted intracellular distribution and tissue expression [1].

An α-GalCer response of rat primary cells has already been shown by us [13]. Here we demonstrate the CD1d dependence of such a response, since the WTH-1 and WTH-2 monoclonal antibodies efficiently inhibited the α-GalCer response of rat and mouse primary cells. These results are consistent with WTH-1 recognizing an epitope in the loop connecting the α1 and the α2 domains of the CD1d molecule, as determined by our mapping studies. According to the CD1d/α-GalCer/TCR crystal structure, this loop is located at the side of the CD1d molecule, where the type I NKT TCR binds α-GalCer-loaded CD1d molecules [44]. Both mAbs also inhibited the response of the type II NKT cell hybridoma VIII24.1 to endogenous antigen presented by a CD1d-transduced B cell tumor. Interestingly, the mAbs exerted opposite effects on the CD1d-dependent recognition of endogenous ligands by another type II NKT cell hybridoma (XV19.2). mAb WTH-2 inhibited the response, while WTH-1 increased the cytokine release. This variability is in line with previous antibody-inhibition experiments [21] and with studies demonstrating the variable response of type II NKT TCRs to point mutants of CD1d [21], [33], [45]. We speculate that the mAb WTH-1 could mediate clustering of the CD1d/ligand complexes on the presenting cell facilitating the formation or improving the function of the immunological synapse on the XV19.2 responder cell, while it may interfere with ligand recognition in case of type I NKT TCRs or the TCR of the other type II NKT-cell hybridoma tested.

The very efficient inhibition of CD1d-dependent antigen recognition by type I NKT cells has some additional implications since it will allow, for the first time, an analysis of CD1d-dependent T cell responses in the rat. This is especially important due to the extended use of the rat a as model organism for a multitude of biological functions and pathological conditions including many autoimmune diseases. In many cases, experiments with mice as well as observations in humans have provided evidence for an involvement of CD1 and CD1d restricted immune responses in these pathological conditions. Now our antibodies do not only allow testing of these parameters in the rat e.g. by blocking CD1d-restricted T cell responses, but also permit a direct comparison of mouse and rat. Furthermore, since the antibodies have been generated in mice, they are ideally suited for *in vivo* studies in this species as they will not provoke a response against xenogenic Ig. Finally, it will be interesting to test whether augmented CD1d autoreactivity of type II NKT TCRs by the mAb WTH-1 as seen with the XV19.2 hybridoma, can also be seen with primary type II NKT cells, as this may allow definition of a subset of type II NKT cells with unique antigen specificity.

## Materials and Methods

### Animals

Animals were kept in cages with filter covers under specific pathogen free conditions (SPF) in line with FELASA recommendations of 2002. The procedures for performing animal experiments as well as animal care were in accordance with the principles of the Bavarian state regulations and approved by the Regierung von Unterfranken (Würzburg, Germany). Animals were used at 6–12 weeks of age. C57BL/6J, BALB/c, C57BL/6 CD1d^−/−^ and BALB/c CD1d^−/−^ mice, as well as LEW/Crl and F344/Crl rats were bred in the animal facilities of the Institute for Virology and Immunobiology, University of Würzburg, Würzburg, Germany. Breeding pairs for CD1d^−/−^ mice, previously bred on a BALB/c background were a kind gift of Heidrun Moll, Institute for Infection Biology, Würzburg University. C57BL/6 CD1d^−/−^ [46] were kindly provided by Mandfred Lutz, Institute for Virology and Immunobiology, University of Würzburg, Würzburg, Germany. BN/SsNOlaHsd, DA/OlaHsd, PVG/OlaHsd, BUF/SimRijHsd, AGUS/OlaHsd, AUG/OlaHsD and WF/NHsd rats were purchased from Harlan laboratories. BH/Ztm rats and CBA/N mice were provided by Kurt Wonigeit, Medical School Hannover, Hannover, Germany. In particular, the analysis of CD1d distribution by immunohistochemistry was carried out on three male LEW/Ztm rats aged about three months and on various female mice: three C57BL/6J female mice aged 8–10 weeks, one BALB/c mouse aged 8–10 weeks and one CBA/N mouse aged about 6 months. One 11 week-old C57BL/6 CD1d^−/−^female and one 10 week old BALB/c CD1d^−/−^ female were used as controls.

### Generation of anti-rat CD1d monoclonal antibodies

BABL/c CD1d^−/−^ mice were immunized i.p. 5 times in weekly intervals with 1×10^7^ CD1d-transduced (F344 allele) M12.4.1.C3 cells. Three weeks after the last immunization, animals were boosted i.v. and after 3 days, splenocytes were fused with Sp2/0 myeloma cells using poylethylene glycol and standard procedures. CD1d specificity of hybridoma culture supernatants was tested by staining mixtures of rat CD1d-transduced and untransduced Raji or P80 [13] cells and primary CD1d positive rat cells. Culture supernatants from 5 hybridomas (WTH-1 (233), 232, WTH-2 (35), WTH-3 (58), and 244) specifically stained rat CD1d transduced cells and rat primary cells. WTH-1, WTH-2 and 232 also stained mouse primary cells. The heavy and light chains of all antibodies were IgG2a and kappa, respectively, as determined by the isotyping test kit (MMT1) from AbD Serotec. Additional sequencing of heavy chain RT-PCR products of hybridomas revealed identical sequences for 232 and WTH-1 on the one hand and for WTH-3 and 244 on the other hand. RT-PCR was performed according to [47] but using the slightly modified degenerate forward primer mMH2 (5′-SARGTNMAGCTGSAGSAGTCWGG-3′) and the reverse primer IgGrev (5′-CAGACTGCAGGAGAGCTGG-3′). Antibodies were purified by Protein A affinity chromatography from culture supernatants using standard procedures. Concentrations were calculated by photometry using the formula: A_280_ × 1.46 - A_260_ × 0.74 = c (mg/ml), where A is absorbance and c is concentration. Purity was determined by SDS-PAGE and Coomassie Blue R250 staining. Fluorescein isothiocyanate (FITC) labeling and biotinylation was performed using standard procedures. FITC from Sigma was incubated at a 20–30 fold molar excess with 1–3 mg/ml purified antibody in sodium (bi)carbonate buffer (0.1 M pH 9.5) for 2 h. FITC was removed by gel filtration through a PD10 column (Amersham Buchler) and exchanged against PBS. Biotinylation was done with Sulfo-NHS-LC-Biotin (Pierce) for 1 h in 0.1 M sodium borate buffer pH 8. The reaction was stopped by addition of TrisHCl (pH 7) and free biotin was removed by centrifugation through ultrafiltration devices such as centricon 30.

### Cloning, mutagenesis and expression of CD1d

Rat CD1d cloned into the pczCFG5 IEGZ retroviral expression vector has been described elsewhere [13]. *Mus musculus* CD1d1 cDNA was sub-cloned into the pczCFG5 IEGN retroviral vector after EcoRI digestion from mCD1d-pczCFG5 IZ [13]. *Mus musculus* CD1d2 and *Mus spretus* CD1d1 were cloned into the EcoRI and BamHI sites of pczCFG5 IEGN [48]. Vectors containing the cDNA sequences used as templates for PCR amplification are described elsewhere [18], [33]. PCR was carried out using the mCD1d-EcoRI-Fow (5′-GGGGAGAATTCCGGCGCTATGCGGTACCTACC-3′) and msCD1d-BamHI-Rev (5′-GCATGGATCCTCACCGGATGTCTTGATAG-3′) primers. Human CD1d cDNA was cloned into the EcoRI and BamHI sites of the pczCFG5 IEGN retroviral vector after RT-PCR using RNA from peripheral blood mononuclear cells as template and the specific primers: hCD1d-MfeI-Fow (5′-AATTCAATTGCGGCGCTATGGGGTGCCTGCTGTTTCTG-3′) and hCD1d-BamHI-Rev (5′-AATTGGATCCTCACAGGACGCCCTGATAGG-3′). The PCR product was digested with MfeI and BamHI. The sequence obtained was the same as the human CD1d mRNA sequence published under the accession number NM_001766.

mCD1d1-D93A and mCD1d1-M162T mutants were generated by combining PCR products as described in Ref. [49]. The inner primers: mCD1d_D93A_Fow (5′-CACCTAAAGAAGCCTATCCCATTG-3′) and mCD1d_D93A_Rev (5′-CAATGGGATAGG CTTCTTTAGGTG-3′) were used for the production of mCD1d1-D93A and mCD1d_M162T_Fow (5′-CGTGCAGACGCTCCTGAATG-3′) and mCD1d_M162T_Rev (5′-CATTCAGGAGCGTCTGCACG-3′) were used for the production of mCD1d1-M162T. In both cases the outer primers used were mCD1d-EcoRI-Fow and mCD1d1-BamHI-Rev (5′-GGGGATCCAAGAGTCACCGGATGTCTTGATAAGGG-3′). Final PCR products were cloned into the EcoRI and BamHI sites of pczCFG5 IEGN vector.

Two chimeras of mouse CD1d1 and human CD1d were produced by PCR of overlapping mouse or human CD1d fragments with the respective primers covering the 5′ or 3′ end of mouse CD1d1 or human CD1d (sequences are given above). Mouse 5′ and 3′ fragments were obtained after EcoRI/BsaI and BsaI/BamHI digestions, respectively. The human fragments were generated with human CD1d as template and the primers hCD1d-MfeI-Fow and T399C-hCD1d-BsaAI-Rev (5′-GAAATGCTACGTGGAAGAAG-3′) for the 5′ fragment and the primers hCD1d-T399-BsaAI-Fow (5′-CTTCTTCCACGTAGCATTTC-3′) and hCD1d-BamHI-Rev for the 3′ fragment. The final PCR products containing the chimeric cDNAs were cloned into the EcoRI and BamHI sites of the expression vector after digestion with EcoRI/BamHI and MfeI/BamHI in case of mouse-5′/human-3′ and human-5′/mouse-3′ chimeras, respectively. All CD1d molecules were transduced into Raji cells using retroviral particles produced as previously described [50]. Transduced cells were sorted using a FACSVantage (BD Biosciences).

### Analysis of rat CD1d alleles

Using genomic DNA from 10 different inbreed rat strains (see animals) as templates, the exons 1 to 3 of CD1d were amplified by PCR with gCD1d_fo (5′- CAAGGGGAGTTGGCTTTGTA-3′) and gCD1d_re (5′-GTGGAGAACCAGGGTGAAAA-3′) primers and sequenced with gCD1d_fo and gCD1d_seq (5′-GCCTGCCACTTCTCAAGC-3′). Additionally, CD1d cDNAs derived from 4 rat strains (LEW/Crl, PVG/OlaHsd, DA/OlaHsd, and BN/SsNOlaHsd) were cloned into pczCFG5 IZ and sequenced as previously described for F344 [13].

### Cell preparation, cell lines and culture

The following cell lines were used either untransduced or transduced with CD1d or control vectors, respectively [13]: P80, CD80 transduced mouse mastocytoma P815; LBB3.4.16, mouse B-cell/B-cell lymphoma hybrid line; M12.4.1.C3, MHC class II deficient variant of the BALB/c M12 B-lymphoma; Sp2/0, HAT sensitive non-secreting mouse myeloma line (ATCC); Raji, Human B-cell lymphoma (ATCC); VIII24.1 and XV19.2, T-cell hybridomas derived from mouse type II NKT cells [14]. Thymocytes and splenocytes were isolated by passing the organ through a metal sieve followed by washing with BSS, 0.2% BSA. Remaining erythrocytes were removed by lysis with TAC buffer (Tris-ammonium chloride, 20 mM Tris (pH 7.2), 0.82% NH4Cl). Primary cells and cell lines were cultured at 37°C with 5% CO_2_ and H_2_O-saturated atmosphere in RPMI 1640 (Invitrogen Life Technologies) supplemented with 5 or 10% FCS, 1 mM sodium pyruvate, 2.05 mM glutamine, 0.1 mM nonessential amino acids, and 5 µM 2-mercaptoethanol (Invitrogen Life Technologies).

For stimulation assays of primary splenocytes, 10^7^ cells/ml were cultured for 24 hours in 96 well plates. α-GalCer (Alexis Biochemicals) and β-GalCer (Sigma) were diluted in DMSO. In CD1d-blocking experiments, purified antibodies or isotype-specific controls were added to the culture at a final concentration of 3.6 µg/ml. Blocking of CD1d antigen presentation to the VIII24.1 hybridoma was analyzed by co-culturing 5×10^4^ mCD1d1 LBB3.4.16 transductants with 5×10^4^ VIII24.1 hybridoma cells for 16 hours with different concentrations of the anti-CD1d mAbs or isotype-specific controls. Blocking of CD1d antigen presentation to the XV19.2 hybridoma was tested with 5×10^4^ mCD1d1 transduced Raji cells as stimulators and 1×10^4^ XV19.2 hybridoma cells as responders. Cultures were carried out in 96 well plates. Cytokine production was quantified using commercial ELISA kits from BD Biosciences (mouse IL-2 and IL-4 and rat IL-4).

### Statistical analysis

In order to analyze the effects of the WTH-1 and WTH-2 mAbs, statistical significance was determined by two-tailed paired Student's t test between cultures with isotype control antibody and WTH-1 or WTH-2 mAbs. A p value of less than 0.05 was considered statistically significant. To analyze the effect of the mAbs or the isotype control antibody on IL-4 release by primary splenocytes cultured with media alone, α-GalCer, β-GalCer or Con A, one-way ANOVA was conducted. p values smaller than 0.05 were considered to be statistically significant. All analyses were performed with the statistical software GraphPad Prism.

### Immunofluorescence and flow cytometry

In binding studies, 1–2×10^5^ Raji transductants or 5×10^5^ thymocytes were incubated in 100 µl FACS buffer (PBS pH 7.4, BSA 0.1%, 0.01% NaN_3_) and indicated concentrations of antibody for 1 hour at room temperature. Subsequently, cells were washed twice with FACS buffer, incubated with phycoerythrin (PE)-labeled donkey anti-mouse IgG (H+L) with minimal cross-reaction to rat and other species serum proteins (Dianova) for 30 minutes (min) at 4°C, washed again and analyzed. Unspecific binding was determined using isotype-matched specific controls (mouse IgG2a).

For competition experiments, 100 µl of cell suspension (1–2×10^5^ Raji transductants or 5×10^5^ thymocytes) were first incubated with 2 µg of unconjugated antibody. After 1 hour of incubation, cells were washed. Then, 10 µl of appropriately diluted biotinylated or PE labeled antibody were added, incubated for a further 30 min at 4°C before cells were washed. Binding of the biotinylated antibodies was revealed by 20 min incubation with streptavidin PE-Cy5 (BD Biosciences) at 4°C. In experiment 2, unspecific inhibition was determined by running parallel samples which were pre-incubated with IgG2a. After a final wash, cells were analyzed on a FACScan or a FACSCalibur using CellQuest software (BD Biosciences).

For the analysis of CD1d expression on different primary rat and mouse cell populations, 10^6^ cells were initially diluted in 100 µl FACS buffer. When mouse derived cells were stained, unspecific binding to mouse Fc receptor was first blocked using the 2.4G2 mAb. Combinations of antibodies used in each staining, are described in the figure legends.

Unless otherwise indicated, antibodies were purchased from BD Biosciences. When mouse cells were analyzed, the following antibodies were used: APCy or FITC anti-TCRβ chain (H57-597), APCy or PE-Cy5 anti-CD4 (RM4), FITC anti-CD21 (7G6), PE anti-CD23 (B3B4), APCy anti-CD19 (1D3) and APCy anti-CD8 (53.6-7). To stain rat cells, the antibodies used were: biotin (bio) or FITC anti-TCRβ (R73), PE, PE-Cy5 or APCy anti-CD4 (OX-35), PE anti-CD4 (OX-38), bio anti-CD8β (341), PerCP anti-CD8α (OX-8), unconjugated dendritic cell marker (OX-62), PE anti-CD11b/c (OX-42), bio or FITC anti-CD45RA (OX-33), bio HIS57, FITC anti-IgM (G53-238) and bio anti-IgD (MARD-3) purchased from AbD Serotec. For the analysis of CD1d, the WTH-1 and WTH-2 antibodies were harvested from hybridomas, purified and aliquots were conjugated or not to biotin or FITC. PE labeled anti-mouse CD1d mAb 1B1 [20] was purchased from BD Biosciences. Anti-human CD1d mAb CD1d42 [34] was a kind of gift of Ekkehard Kämpgen, Dermatology Dept. University of Erlangen. Detection of biotinylated antibodies was performed with streptavidin (SA) labeled with PE, PE-Cy5 or APCy from BD Biosciences. When mouse derived unconjugated antibodies were used in multicolor stainings, samples were first incubated with the unconjugated antibody only, then washed and bound antibody was detected by incubation with a FITC or PE labeled donkey F(ab')_2_ fragment anti-mouse IgG (H+L) with minimal cross-reaction to rat and other species serum proteins (Dianova). After a further washing step, unspecific binding to the donkey anti-mouse reagent during subsequent stainings was blocked by adding substantial amounts of serum mouse IgG for 15 min. Flow cytometry was carried out using a FACSCalibur (BD Biosciences) and data were analyzed with CellQuest (BD Biosciences) and FlowJo software (Tree Star).

### Immunoprecipitation

Thymocytes and transduced cell lines were biotinylated with 2 mg/ml Sulfo-NHS-LC-Biotin (Pierce) in PBS for 15 min on a rotating platform at 4°C and washed twice with RPMI (supplemented as for cultures) followed by two additional washing steps with BSS/BSA. After biotinylation 10^6^ transductants or 5×10^7^ cells thymocytes were lysed in 1 ml of lysis buffer I (50 mM Tris, pH 7.4, 150 mM NaCl, 0.25% Na-deoxycholate, 1 mM EDTA, 1% NP40 and protease inhibitors (Complete Mini, Roche)) for 30 min on ice. Nuclei were removed by pelleting immediately after lysis and the lysate was pre-absorbed one hour on Protein A Sepharose (GE, Healthcare) at 4°C. For specific immunoprecipitation, Protein A Sepharose beads were pre-coated with rabbit anti-mouse IgG (ICN Biomedicals) before the addition of monoclonal antibodies. Then, the matrices were covalently linked as previously described [51]. Pre-absorbed lysates were incubated overnight with specific immunomatrices. Precipitates were then washed four times with lysis buffer and were resuspended in loading buffer (2% SDS, 62.5 mM Tris pH 6.8, 10% glycerol, 770 mM 2-mercaptoethanol and 0.04% bromophenol blue) and boiled for 5 min before loading onto SDS-PAGE. The molecular weight marker used, PageRuler™ Prestained Protein Ladder, was purchased from Fermentas. After SDS-PAGE and blotting, membranes were incubated with streptavidin-horseradish peroxidase (HRP) (BD Biosciences) and detection was carried out by ECL (GE, Healthcare).

### Western blot analysis

Protein preparation from rat tissues was performed by disrupting the tissues with a rotor-stator homogenizer directly into lysis buffer II (1% NP40, 0.5% Na-deoxycholate, 0.1% SDS in PBS) supplemented with protease inhibitors (Complete Mini, Roche). After protein concentrations had been determined by using the Bio-Rad protein assay (Bio-Rad), 20 µg of protein were mixed with non-reducing loading buffer (4x concentrated: 250 mM Tris-HCl pH 6.8, 8% SDS, 40% glycerol and 0.16% bromophenol blue) and were separated by 10% SDS-PAGE. For the analysis of cell lines expressing CD1d, whole cell lysates (2×10^7^ cells in 1 ml lysis buffer II) were mixed with non-reducing loading buffer and 18 µl were separated by 10% SDS-PAGE. The molecular weight marker used, PageRuler™ Prestained Protein Ladder, was purchased from Fermentas. Proteins were transferred to a Roti-polyvinylidene difluoride membrane (Roth). For CD1d detection, WTH-1 and WTH-2 mAbs were used at a final concentration of 0.75 µg/ml. As protein loading control anti-ERK2 (C-14, sc-154) rabbit polyclonal antibody (Santa Cruz Biotechnology) was used at a final concentration of 2 µg/ml. Primary antibodies were detected with HRP-conjugated goat anti-mouse antibody (Santa Cruz Biotechnology). All antibodies were diluted in PBS, 0.1% Tween and 2.5% non-fat dried milk (AppliChem). Films were developed by chemoluminescence using the ECL Developing kit (GE Healthcare).

### Immunohistology

Cryosections were stored at −20°C overnight and immediately fixed in 100% isopropanol for 10 min at 4°C. After washing in PBS, the sections were treated with a solution of 10 mM glucose, 1 mM NaN_3_ and 900 U/ml glucose oxidase in PBS for 45 min at 37°C to block endogenous peroxidase activity. Lymphatic organs, pancreas, liver and gut sections were then preincubated with 0.003 mg/ml avidin in PBS/BSA/NaN_3_ for 20 h at 4°C to avoid background staining due to endogenous biotin. After washing in PBS, biotinylated WTH-1 or WTH-2 antibodies diluted 1∶200 in PBS/BSA/NaN_3_ containing a final concentration of 0.02 mg biotin/ml were applied for 20 h at 4°C. After one further wash in PBS the sections were incubated with the avidin-biotinylated peroxidase complex (ABC) of the Vectastain ABC elite kit (Vector Laboratories) according to the recommendations of the manufacturer by applying the AB-solution at 1∶50 in PBS for 30 min at room temperature. After washing in Tris-buffered saline, a standard diaminobenzidine reaction was carried out. Half of the sections were counterstained with Mayer's hemalum. The sections were then dehydrated in increasing concentrations of isopropanol with two final exchanges of xylene and coverslipped in Eukitt. Control sections without antibody were always included in the assay.

With the exception of the thymus, mouse organs were stained using a tyramide amplification technique, because the staining intensity in mouse parenchymal cells was reduced compared to rats. Biotinylated tyramide had been prepared before by incubating 50 mg Sulfo-NHS-LC-biotin (Pierce) in 20 ml 0.025 borate buffer pH 8.5 with 15 mg tyramine-HCl (Sigma) on a stirrer overnight. After filtration this solution was aliquoted and frozen. For application aliquots were thawed and diluted 1∶1000 in Tris-buffered saline pH 7.6 containing 0.09 mM H_2_O_2_. Antibody incubation was performed as described above. After the first incubation with the ABC the sections were washed and covered with biotinylated tyramide/H_2_O_2_ for 10 min at room temperature. After a further washing step the ABC was applied, again followed by the diaminobenzidine reaction. By this procedure the peroxidase in the first AB-complexes catalyzes the binding of biotinylated tyramide to proteins on the surface of the section, thus, substantially increasing the amount of biotin available for a second reaction with the ABC.

Immunohistology was also performed on rat organ cryosections by incubating unconjugated antibodies overnight at the same dilutions as for the staining with biotinylated antibodies in a standard ABC-immunoperoxidase system (Vectastain ABC elite kit, Vector Laboratories). In this case, 0.003 mg/ml avidin was directly added to the primary antibody and 0.02 mg/ml biotin was included in the secondary anti-mouse reagent. Both staining methods gave identical results. W3/25 and HIS57 were purchased from AbD Serotec and BD Biosciences, respectively. W3/25 was used at a dilution of 1∶200 and HIS57 at a dilution of 1∶500 followed by the ABC detection system mentioned above.

## Supporting Information

Figure S1 CD1d cell surface expression in five different rat strains. CD1d cell surface expression was analyzed by flow cytometry using the biotinylated WTH-1 mAb visualized with SA-APCy in thymocytes and splenocytes derived from F344, PVG, BN, LEW and DA rats.
(0.21 MB TIF)

Figure S2 CD1d expression by B and T cells analyzed by multicolor flow cytometry. T and B cells were identified using antibodies visualized with APCy in order to avoid unspecific signal due to fluorescence spectral overlap in the PE channel used for CD1d detection. Therefore, CD1d was analyzed in separate multicolor experiments with same overall CD1d staining intensity (Number 2 histograms: gray and black lines correspond to B- and T-cell stainings, respectively). Number 1 dot plots show gates on total splenocytes which were further analyzed. For CD1d detection in C57BL/6 mice, biotinylated WTH-2 mAb followed by SA-PE was used. In rats, CD1d was detected with unconjugated WTH-2 mAb followed by PE-labeled donkey anti-mouse IgG. Number 4 dot plots indicate coexpression of CD1d and T or B cell markers. Number 3 dot plots represent isotype control stainings for CD1d mAbs. Gray and black dot plots are B- and T-cell stainings, respectively. In mouse, B cells were stained with anti-CD19 (1D3-APCy) mAb and T cells with anti-TCRβ chain mAb (H57-597-APCy). In rat, B cells were defined as CD45RA (OX-33-biotin + SA-APCy) positive cells and for the identification of T cells, anti-TCRβ chain (R73-biotin + SA-APCy) antibody was used. Boxes indicate gated cells shown in number 5 histograms and numbers inside the plots correspond to the percentages of gated cells. These histograms are also shown in figure 4C. The data shown are one representative of three experiments performed.
(1.74 MB TIF)

Figure S3 Relative CD1d expression by CD4 and CD8 positive T cells analyzed by multicolor flow cytometry. Dot plots on the left show gated splenocytes which were studied. CD4 and CD8 positive T cell gating strategies are illustrated with the histograms and dot plots in the middle columns. As indicated in the labelling of the axes, two different antibody combinations were used to stain LEW cells: one with biotinylated WTH-2 mAb visualized with SA-PE (upper row) and other with unconjugated WTH-2 mAb detected with PE-labeled donkey anti-mouse IgG (DαM-PE, lower row). Numbers in the gated plots indicate percentages of gated cells. Fluorescence 2 MFI of CD4 and CD8 T cells in stainings, where instead of the anti-CD1d mAb an isotype control Ab was used, were: in the staining of C57BL/6 cells, 5.98 and 4.58 in CD4 and CD8 gated cells respectively; in the upper LEW staining: 10.2 and 10.6 for CD4 and CD8 T cells, respectively, and in the lower staining of rat cells: 5.32 for CD4, and 5.09 for CD8 positive T cells. In the histograms on the right, gray and black lines correspond to CD4 and CD8 positive T cells, respectively. Filled histograms are control stainings. In the figure 4C the lowest histogram of rat T cells and the histogram of mouse T cells are shown. One representative of three experiments is shown.
(1.11 MB TIF)

Table S1 Rat CD1d alleles. Two different rat CD1d alleles were identified in this study and were compared to a previous report and to the rat BN genome. Analysis of copy DNA (cDNA) of BN, LEW, F344, PVG and DA rats as well as of genomic DNA covering the exons 1 to 3 of these strains and also of BUF, BH, AGUS, AUG and WF rats was carried out as described in the methods section. A single nucleotide substitution encoding a phenylalanine instead of a valine in the exon 1 (position −4 of the mature peptide) was found only in F344 rats. The sequence found in the BN genome at the NCBI is identical to the BN allele found in this study. A previous report (Katabami et al., 1998), where the exon 1 was only analyzed for F344 rats, reported a nucleotide substitution in exon 3 which would encode an alanine in seven and a valine in five rat strains. In contrast, the CD1d nucleotide sequences of all the strains analyzed by us and the BN genomic sequence at the NCBI, encode an alanine at this position. Katabami, S., Matsuura, A., Chen, H.Z., Imai, K., and Kikuchi, K. (1998). Structural organization of rat CD1 typifies evolutionarily conserved CD1D class genes. Immunogenetics 48, 22–31.
(0.03 MB DOC)

Table S2 CD1d in rat non-lymphatic organs (mAbs WTH-1 and WTH-2).
(0.03 MB DOC)

Table S3 CD1d in mouse non-lymphatic organs (mAb WTH-1). * only some apical granules. ** only apical granules in few cells.
(0.03 MB DOC)

Table S4 IL-4 production by mouse and rat splenocytes. Splenocytes derived from C57BL/6 mice or F344 rats were cultured in the presence or absence of mAbs with media alone, α-GalCer (10 ng/ml), β-GalCer (10 ng/ml) or Con A (2 µg/ml) for 24 hours and IL-4 secretion into the supernatants was determined by ELISA. Mean values (pg/ml) ±SD obtained from three independent experiments are shown. To asses the effects of the antibodies in each culture condition (media only, α-GalCer, β-GalCer or Con A) one-way ANOVA was conducted. Significant differences were only obtained in α-GalCer cultures where the p values obtained were 0.02 and 0.0007 for rat and mouse, respectively.
(0.03 MB DOC)
