# Supplementary figures and images for: CD1d Expression in Paneth Cells and Rat Exocrine Pancreas Revealed by Novel Monoclonal Antibodies Which Differentially Affect NKT Cell Activation
Source: PLoS One. 2010 Sep 30;5(9):e13089. doi: 10.1371/journal.pone.0013089 (PMC2948036; doi:10.1371/journal.pone.0013089)

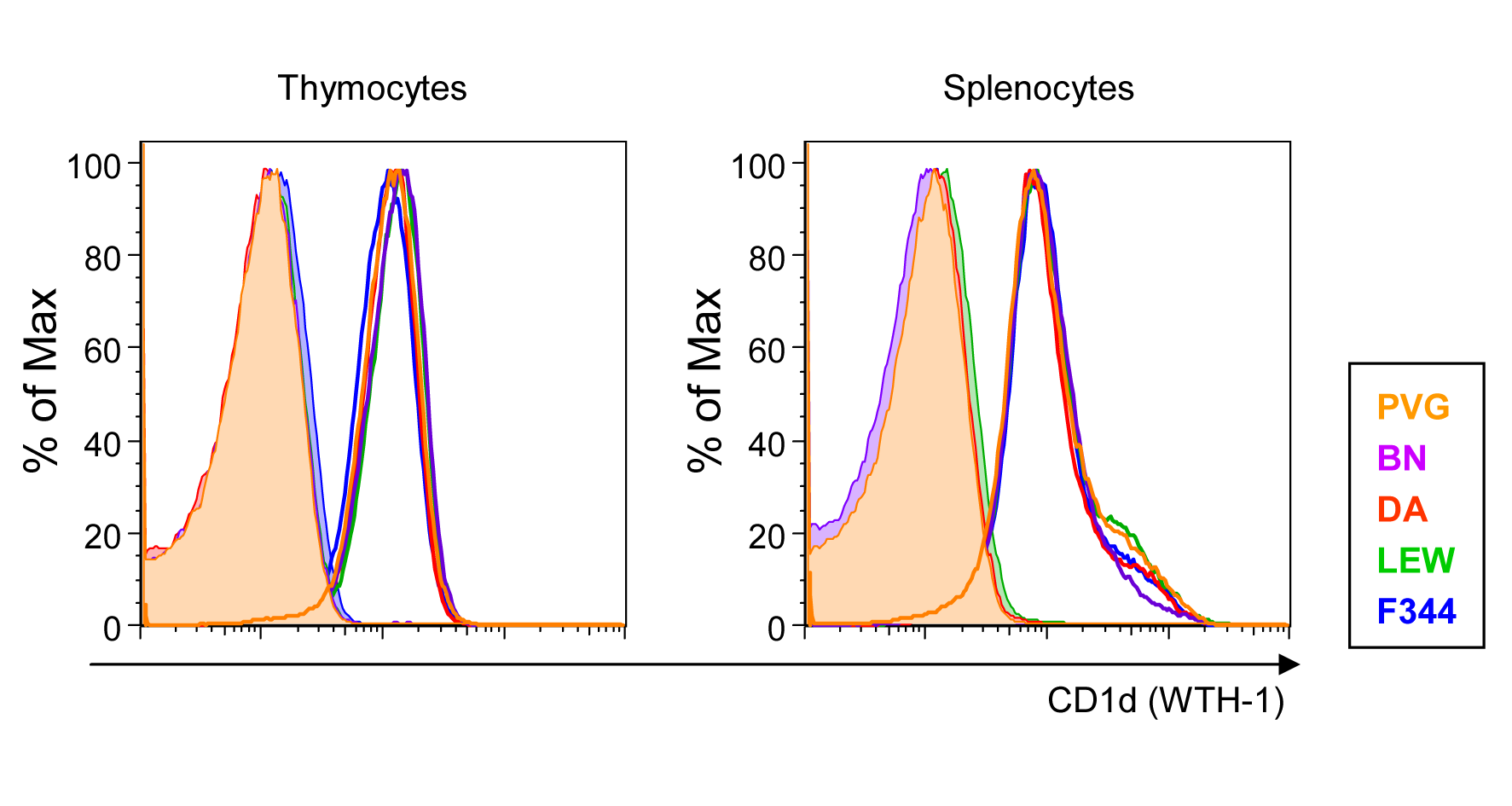

Supplement: Figure S1 — CD1d cell surface expression in five different rat strains. CD1d cell surface expression was analyzed by flow cytometry using the biotinylated WTH-1 mAb visualized with SA-APCy in thymocytes and splenocytes derived from F344, PVG, BN, LEW and DA rats. (0.21 MB TIF) [file pone.0013089.s001.tif]

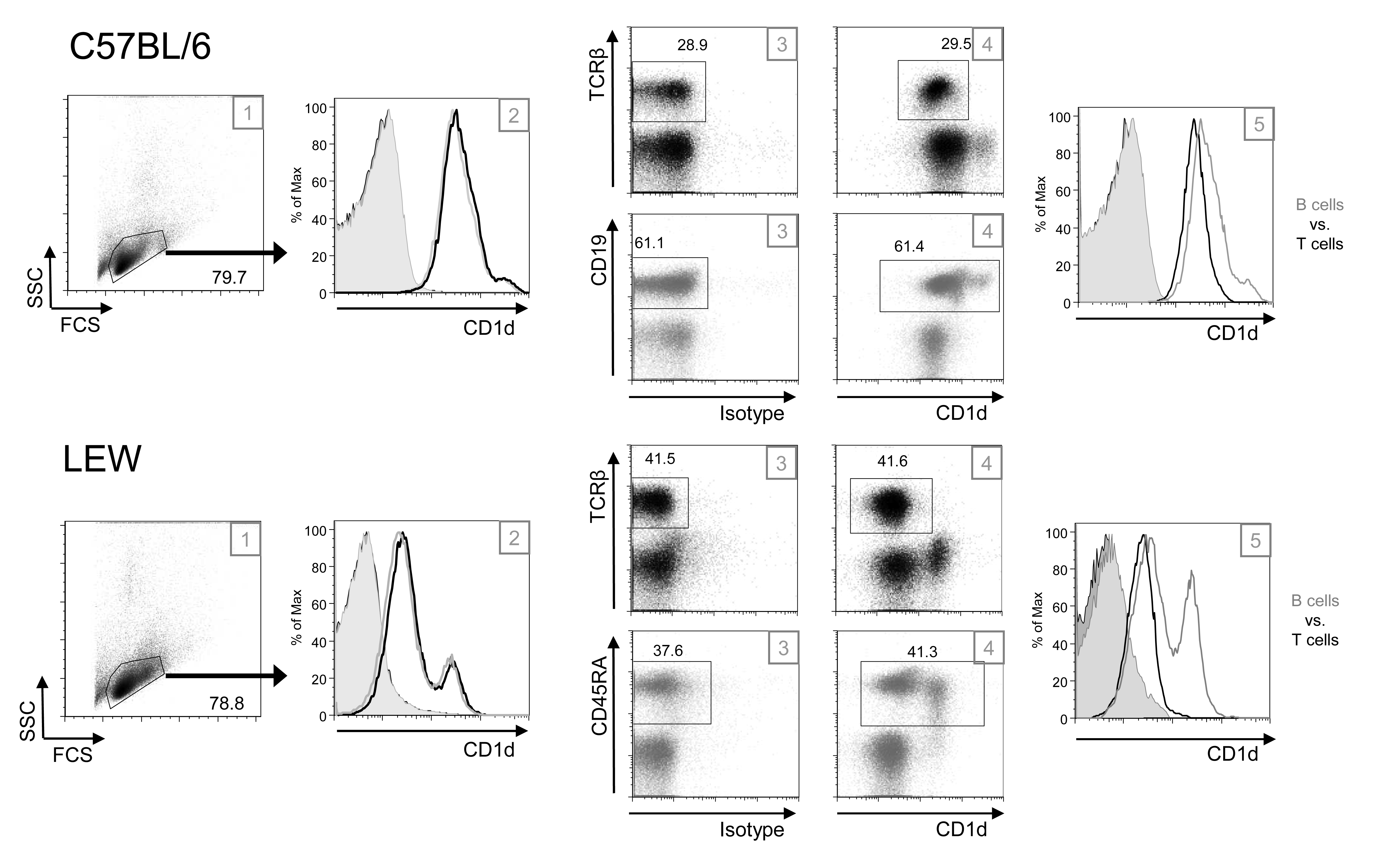

Supplement: Figure S2 — CD1d expression by B and T cells analyzed by multicolor flow cytometry. T and B cells were identified using antibodies visualized with APCy in order to avoid unspecific signal due to fluorescence spectral overlap in the PE channel used for CD1d detection. Therefore, CD1d was analyzed in separate multicolor experiments with same overall CD1d staining intensity (Number 2 histograms: gray and black lines correspond to B- and T-cell stainings, respectively). Number 1 dot plots show gates on total splenocytes which were further analyzed. For CD1d detection in C57BL/6 mice, biotinylated WTH-2 mAb followed by SA-PE was used. In rats, CD1d was detected with unconjugated WTH-2 mAb followed by PE-labeled donkey anti-mouse IgG. Number 4 dot plots indicate coexpression of CD1d and T or B cell markers. Number 3 dot plots represent isotype control stainings for CD1d mAbs. Gray and black dot plots are B- and T-cell stainings, respectively. In mouse, B cells were stained with anti-CD19 (1D3-APCy) mAb and T cells with anti-TCRβ chain mAb (H57-597-APCy). In rat, B cells were defined as CD45RA (OX-33-biotin + SA-APCy) positive cells and for the identification of T cells, anti-TCRβ chain (R73-biotin + SA-APCy) antibody was used. Boxes indicate gated cells shown in number 5 histograms and numbers inside the plots correspond to the percentages of gated cells. These histograms are also shown in figure 4C. The data shown are one representative of three experiments performed. (1.74 MB TIF) [file pone.0013089.s002.tif]

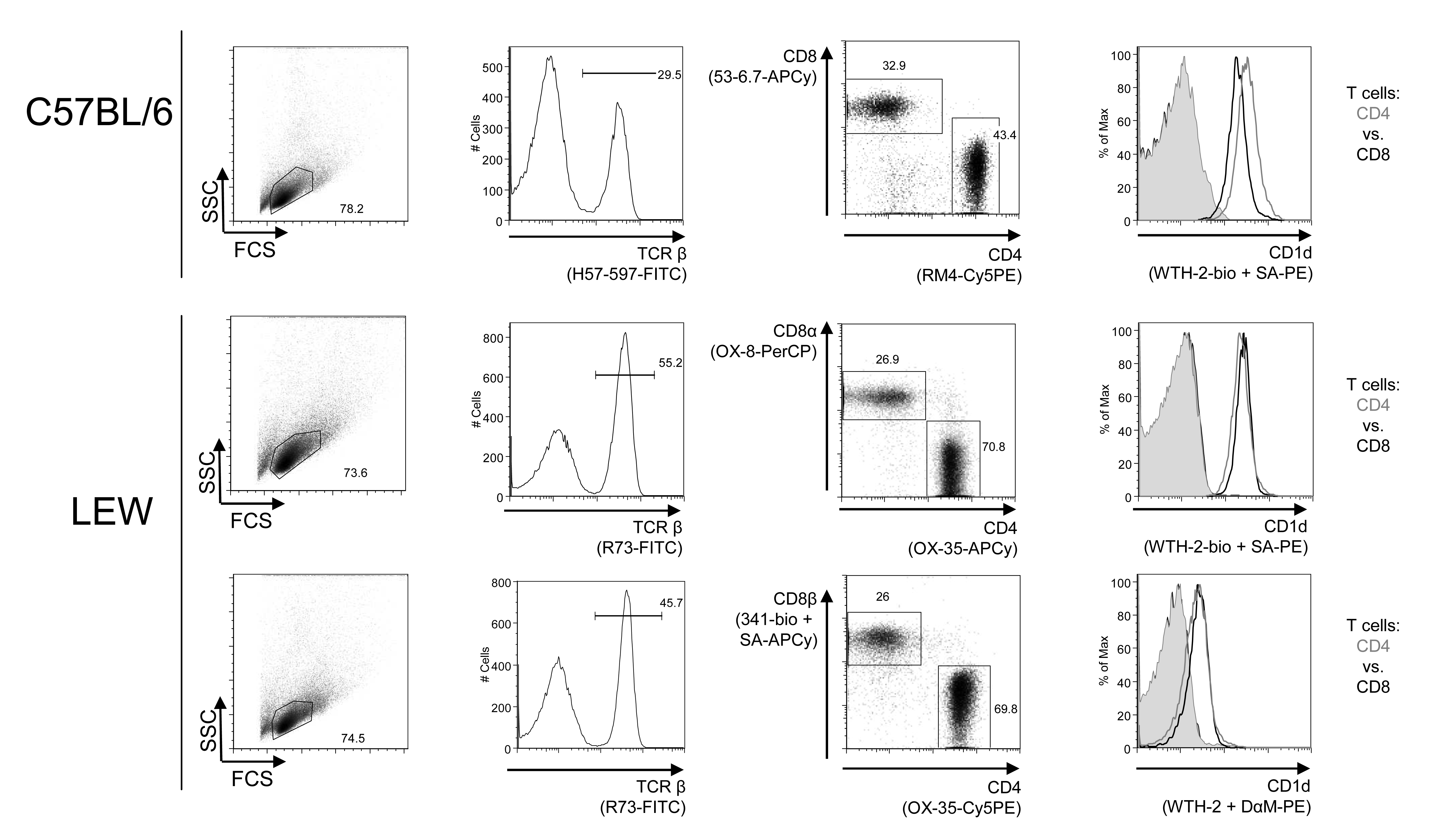

Supplement: Figure S3 — Relative CD1d expression by CD4 and CD8 positive T cells analyzed by multicolor flow cytometry. Dot plots on the left show gated splenocytes which were studied. CD4 and CD8 positive T cell gating strategies are illustrated with the histograms and dot plots in the middle columns. As indicated in the labelling of the axes, two different antibody combinations were used to stain LEW cells: one with biotinylated WTH-2 mAb visualized with SA-PE (upper row) and other with unconjugated WTH-2 mAb detected with PE-labeled donkey anti-mouse IgG (DαM-PE, lower row). Numbers in the gated plots indicate percentages of gated cells. Fluorescence 2 MFI of CD4 and CD8 T cells in stainings, where instead of the anti-CD1d mAb an isotype control Ab was used, were: in the staining of C57BL/6 cells, 5.98 and 4.58 in CD4 and CD8 gated cells respectively; in the upper LEW staining: 10.2 and 10.6 for CD4 and CD8 T cells, respectively, and in the lower staining of rat cells: 5.32 for CD4, and 5.09 for CD8 positive T cells. In the histograms on the right, gray and black lines correspond to CD4 and CD8 positive T cells, respectively. Filled histograms are control stainings. In the figure 4C the lowest histogram of rat T cells and the histogram of mouse T cells are shown. One representative of three experiments is shown. (1.11 MB TIF) [file pone.0013089.s003.tif]
