# Supplementary material for: CD1d Expression in Paneth Cells and Rat Exocrine Pancreas Revealed by Novel Monoclonal Antibodies Which Differentially Affect NKT Cell Activation
Source: PLoS One. 2010 Sep 30;5(9):e13089. doi: 10.1371/journal.pone.0013089 (PMC2948036; doi:10.1371/journal.pone.0013089)
Supplement: Table S1 — Rat CD1d alleles. Two different rat CD1d alleles were identified in this study and were compared to a previous report and to the rat BN genome. Analysis of copy DNA (cDNA) of BN, LEW, F344, PVG and DA rats as well as of genomic DNA covering the exons 1 to 3 of these strains and also of BUF, BH, AGUS, AUG and WF rats was carried out as described in the methods section. A single nucleotide substitution encoding a phenylalanine instead of a valine in the exon 1 (position −4 of the mature peptide) was found only in F344 rats. The sequence found in the BN genome at the NCBI is identical to the BN allele found in this study. A previous report (Katabami et al., 1998), where the exon 1 was only analyzed for F344 rats, reported a nucleotide substitution in exon 3 which would encode an alanine in seven and a valine in five rat strains. In contrast, the CD1d nucleotide sequences of all the strains analyzed by us and the BN genomic sequence at the NCBI, encode an alanine at this position. Katabami, S., Matsuura, A., Chen, H.Z., Imai, K., and Kikuchi, K. (1998). Structural organization of rat CD1 typifies evolutionarily conserved CD1D class genes. Immunogenetics 48, 22–31. (0.03 MB DOC) [file pone.0013089.s004.doc]

**Table S1. Rat CD1d alleles**

|  | Position -4 | | Position 101 | |
| --- | --- | --- | --- | --- |
| Source: | Phenylalanine (TTC) | Valine  (GTC) | Valine  (GTT) | Alanine  (GCT) |
| Monzon-Casanova, E., Pyz, E. and Herrmann, T. | F344/Crl | LEW/Crl,  BN/SsNOlaHsd, DA/OlaHsd, PVG/OlaHsd, BUF/SimRijHsd, AGUS/OlaHsd, AUG/OlaHsD, WF/NHsd |  | LEW/Crl, F344/Crl,  BN/SsNOlaHsd, DA/OlaHsd, PVG/OlaHsd, BUF/SimRijHsd, AGUS/OlaHsd, AUG/OlaHsD, WF/NHsd |
| Katabami et al. (Katabami et al., 1998) | F344/Crj |  | LEW/Crj, Wistar/Crj/Smc,  TO/Hkm, WKAH/Hkm, W/N/Hkm | F344/Crj, NIGIII/Hok, LEJ/Hkm,  ALB/Hkm, SDJ/Hok, ACI/Hkm,  BN/Hok |
| BN genome (NW_047626.2) |  | BN/SsNHsdMCW |  | BN/SsNHsdMCW |
