# Supplementary material for: CD1d Expression in Paneth Cells and Rat Exocrine Pancreas Revealed by Novel Monoclonal Antibodies Which Differentially Affect NKT Cell Activation
Source: PLoS One. 2010 Sep 30;5(9):e13089. doi: 10.1371/journal.pone.0013089 (PMC2948036; doi:10.1371/journal.pone.0013089)
Supplement: Table S2 — CD1d in rat non-lymphatic organs (mAbs WTH-1 and WTH-2). (0.03 MB DOC) [file pone.0013089.s005.doc]

**Table S2. CD1d in rat non-lymphatic organs (mAbs WTH-1 and WTH-2)**

| **liver** |  | **heart** |  | **ileum** |  | **pancreas** |  |
| --- | --- | --- | --- | --- | --- | --- | --- |
| hepatocytes | + | cardiocytes | + | enterocytes | + | acinar cells | ++ |
| sinusoidal endothelia | ++ | endocardium | ++ | enteroendocrine cells | ? | intercalated duct epithelia | ? |
| Kupffer cells | ? |  |  | Paneth cells | ++ | interlobular duct epithelia | + |
| stellate cells | ? |  |  | villous stroma | ++ | endocrine islet cells | +/- |
|  |  |  |  | smooth myocytes (T. muscularis) | - |  |  |
