# Supplementary material for: CD1d Expression in Paneth Cells and Rat Exocrine Pancreas Revealed by Novel Monoclonal Antibodies Which Differentially Affect NKT Cell Activation
Source: PLoS One. 2010 Sep 30;5(9):e13089. doi: 10.1371/journal.pone.0013089 (PMC2948036; doi:10.1371/journal.pone.0013089)
Supplement: Table S3 — CD1d in mouse non-lymphatic organs (mAb WTH-1). * only some apical granules. ** only apical granules in few cells. (0.03 MB DOC) [file pone.0013089.s006.doc]

**Table S3. CD1d in mouse non-lymphatic organs (mAb WTH-1)**

| **ileum** |  | **pancreas** |  |
| --- | --- | --- | --- |
| enterocytes | - | acinar cells** | +/- |
| enteroendocrine cells | + | intercalated duct epithelia | ? |
| Paneth cells* | + | interlobular duct epithelia | + |
| villous stroma | + | endocrine islet cells | - |
| smooth myocytes (T. muscularis) | - |  |  |
