# Supplementary material for: CD1d Expression in Paneth Cells and Rat Exocrine Pancreas Revealed by Novel Monoclonal Antibodies Which Differentially Affect NKT Cell Activation
Source: PLoS One. 2010 Sep 30;5(9):e13089. doi: 10.1371/journal.pone.0013089 (PMC2948036; doi:10.1371/journal.pone.0013089)
Supplement: Table S4 — IL-4 production by mouse and rat splenocytes. Splenocytes derived from C57BL/6 mice or F344 rats were cultured in the presence or absence of mAbs with media alone, α-GalCer (10 ng/ml), β-GalCer (10 ng/ml) or Con A (2 µg/ml) for 24 hours and IL-4 secretion into the supernatants was determined by ELISA. Mean values (pg/ml) ±SD obtained from three independent experiments are shown. To asses the effects of the antibodies in each culture condition (media only, α-GalCer, β-GalCer or Con A) one-way ANOVA was conducted. Significant differences were only obtained in α-GalCer cultures where the p values obtained were 0.02 and 0.0007 for rat and mouse, respectively. (0.03 MB DOC) [file pone.0013089.s007.doc]

**Table S4**. IL-4 production by mouse and rat splenocytes.

|  | Mouse | | | | Rat | | | |
| --- | --- | --- | --- | --- | --- | --- | --- | --- |
|  | - | Isotype | WTH-1 | WTH-2 | - | Isotype | WTH-1 | WTH-2 |
| Media | 2.10 ±0.52 | 3.13 ±0.55 | 2.18 ±1.0 | 2.03 ±0.25 | 0.13 ±0.23 | 0.07 ±0.12 | 0.03 ±0.06 | 0.00 ±0.00 |
| α-GalCer | 21.7 ±6.34 | 23.97 ±9.81 | 3.40 ±2.45 | 2.47 ±2.63 | 6.57 ±3.3 | 6.13 ±4.36 | 0.27 ±0.46 | 0.00 ±0.00 |
| β-GalCer | 1.33 ±1.59 | 1.10 ±1.28 | 1.10 ±1.21 | 1.23 ±1.08 | 0.07 ±0.12 | 0.00 ±0.00 | 0.07 ±0.12 | 0.10 ±0.17 |
| Con A | 8.97 ±2.03 | 13.3 ±5.6 | 10.31 ±4.99 | 8.83 ± 3.71 | 6.80 ±1.66 | 7.20 ±0.66 | 5.63 ±3.58 | 5.37 ±0.98 |
